# Mutation analysis of large tumor suppressor genes *LATS1* and *LATS2* supports a tumor suppressor role in human cancer

**DOI:** 10.1007/s13238-014-0122-4

**Published:** 2014-12-09

**Authors:** Tian Yu, John Bachman, Zhi-Chun Lai

**Affiliations:** 1 Intercollege Graduate Degree Program in Molecular, Cellular and Integrative Biosciences, The Pennsylvania State University, University Park, PA 16802 USA; 2 Department of Biology, The Pennsylvania State University, University Park, PA 16802 USA; 3 Department of Biochemistry and Molecular Biology, The Pennsylvania State University, University Park, PA 16802 USA

**Keywords:** *LATS1* & *LATS2*, hippo signaling, cancer genome, human cancer

## Abstract

In recent years, human cancer genome projects provide unprecedented opportunities for the discovery of cancer genes and signaling pathways that contribute to tumor development. While numerous gene mutations can be identified from each cancer genome, what these mutations mean for cancer is a challenging question to address, especially for those from less understood putative new cancer genes. As a powerful approach, *in silico* bioinformatics analysis could efficiently sort out mutations that are predicted to damage gene function. Such an analysis of human large tumor suppressor genes, *LATS1* and *LATS2*, has been carried out and the results support a role of *hLATS1/*/*2* as negative growth regulators and tumor suppressors.

**Electronic supplementary material:**

The online version of this article (doi:10.1007/s13238-014-0122-4) contains supplementary material, which is available to authorized users.

## Introduction

**Figure 1:**
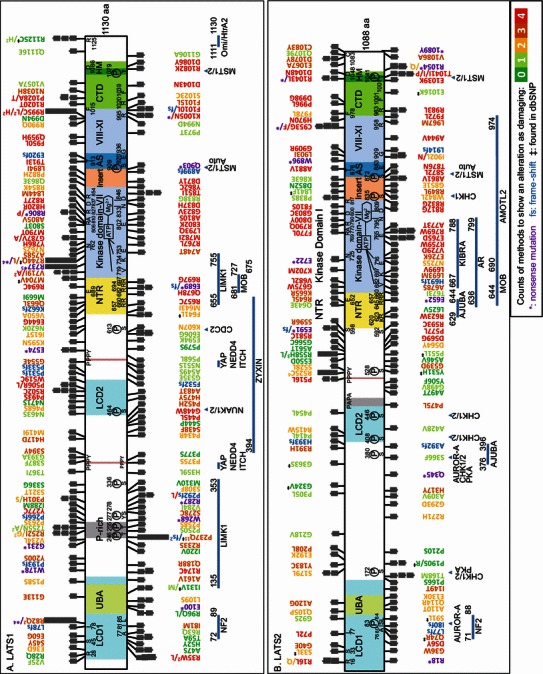
**Human cancer mutations in LATS1 (A) and LATS2 (B) are mapped to their corresponding open reading frames**. Human LATS1 and LATS2 mutation data was collected from Catalogue of Somatic Mutations in Cancer (COSMIC) (top portions) and cBioPortal (bottom portions) databases. (A complete list of all mutations can be found in Table S1). All non-synonymous mutations are analyzed, using Uniprot identifier O95835 for LATS1 and Q9NRM7 for LATS2. Synonymous mutations were not included in this analysis. To evaluate potential impact of individual mutations on LATS1/2 structure and function, the following bioinformatics resources were used: 1) SIFT; 2) PROVEAN; 3) PolyPhen-2; and 4) Mutation Assessor. A color code was used to distinguish mutations that are predicted to be damaging by various numbers of the methods described above (zero in dark green, one in light green, two in orange yellow, three in brown, and four in red). “*” indicates nonsense mutation and “‡” for ones found in dbSNP. “fs” is for frame-shift. Blue bars indicate regions involved in protein-protein interactions as indicated. Blue triangles identify phosphorylation sites by corresponding protein kinases. Each small block square indicates one unique mutation sample for *LATS1*/*2* from human cancer

Hippo signaling plays a crucial role in animal development and tumorigenesis (Harvey et al., 2013; Yu and Guan, 2013). As a key regulator of this growth-inhibitory pathway, the large tumor suppressor (*Lats*)/*warts* (*wts*) gene encodes a Ser/Thr protein kinase and somatic mutations in human *LATS1* and *LATS2* have been identified in primary tumors (e.g. Murakami et al., 2011; Visser and Yang, 2010). To systematically evaluate how *LATS1*/*2* genes play a critical role in human cancer, a mutation analysis has been carried out. In the Catalogue of Somatic Mutation in Cancer (COSMIC) database, 101 non-synonymous *LATS1* somatic mutations have been identified from 9183 unique human tumor samples (Fig. 1A). Similarly, there are 80 *LATS2* non-synonymous mutations out of 9516 samples (Fig. 1B). Therefore, an overall mutation rate is 1.10% for *LATS1* and 0.84% for *LATS2*. In the cBioPortal database, *LATS1* and *LATS2* exhibited similar overall mutation rates, 1.83% (135/7390) and 1.50% (111/7390), respectively. The top three highest mutation rates with relative larger total sample size for *LATS1* occurred in stomach adenocarcinoma (5.91%, *n* = 220), uterine corpus endometrial carcinoma (4%, *n* = 248), and bladder urothelial carcinoma (3.1%, *n* = 130). Meanwhile, the highest *LATS2* non-synonymous mutation rate occurred in uterine corpus endometrial carcinoma (5.2%, *n* = 248), stomach adenocarcinoma (4.1%, *n* = 220), and lung adenocarcinoma (3.9%, *n* = 229) (Table S1).

To determine the mutation distribution across different domains, analysis through Fisher’s exact test shows that both the kinase domain (*P* = 0.01075) and proline-rich (*P* = 0.0312) of LATS1 displayed the highest mutation frequency among all the LATS1 domains. The proline-rich domain had 7 mutations in a 31-amino acid (aa) region (2.2 mutations/10 aa), and the kinase domain had 43 mutations in a 306-aa region (1.41 mutations/10 aa) (Fig. 1A). In LATS2, the kinase domain (*P* = 5.66 × 10^−5^) and insertion domain (*P* = 0.03121) had the highest mutation frequency. LATS2 kinase domain had 44 mutations in a 306-aa region (1.48 mutation/10 aa) and LATS2 insertion domain had 9 in 45 aa (2 mutation/10 aa) (Fig. 1B). These data support that selections have occurred to enrich mutations in functionally significant regions such as the kinase domain to facilitate tumorigenesis.

Among all the mutations, nonsense and frame-shift mutations clearly disrupt *LATS1*/*2* gene function. Thirteen and 10 unique nonsense mutations were found in *LATS1* and *LATS2*, respectively. Moreover, 12 and 7 unique frame-shift mutations were detected in *LATS1* and *LATS2*, respectively (Fig. 1). The pattern of equal distribution of *LATS1*/*2* nonsense or frame-shift mutations is consistent with the idea that *LATS1* and *LATS2* are tumor suppressor genes. The percentage of either nonsense or frame-shift mutations among all the mutations for *LATS1* and *LATS2*, was 17.43% and 10.69%, respectively.

To predict the functional relevance of other non-synonymous mutations in LATS1/2, we performed analyses of evolutionary conservation and protein structure through four different mutation-assessing methods: SIFT, PROVEAN, PolyPhen-2, and Mutation Assessor (Ng and Henikoff, 2003; Adzhubei et al., 2010; Reva et al., 2011; Choi et al., 2012). We found that 73.85% (161/218) of missense mutations from *LATS1* and 77.98% (124/159) from *LATS2* are predicted to be damaging by at least one method (Fig. 1). In regards to mutations that were considered severe as determined by all four methods, LATS1 had a percentage of 25.69% (56/218) and LATS2 had 31.44% (50/159).

## LCD1 and LCD2

LATS1/2 proteins share LATS conserved domain 1 (LCD1) and LATS conserved domain 2 (LCD2), which are conserved in all vertebrate LATS1/2 homologues. LCD1 and LCD2 are critical for LATS1/2 function and regulation. The deletions of either LCD1 or LCD2 in mouse Lats2 abolished its tumor suppressor activity in immortalized mouse cell line (Visser and Yang, 2010). Lats1 LCD1 knockout mice were born with a low birth rate, from which the mouse embryonic fibroblasts displayed chromosomal instability and tumorigenesis (Yabuta et al., 2011). Within LCD1, a short segment called Conserved N-terminal Motif (CNM) (aa 72–89 for LATS1 and 71–88 for LATS2) is important for membrane recruitment and activation of LATS1/2 by Merlin/NF2. The alterations of three highly conserved residues in LATS1/2-A77/76P-I81/80T-L85/84P prevented its interaction with Merlin/NF2, membrane localization and activation (Yin et al., 2013). Therefore, LATS1-I81M, LATS1-R82Q, and LATS2-P72L mutations may fail to interact with Merlin/NF2 and consequently cannot be activated (Fig. 1). The truncated products of LATS1 such as L78fs, R82*, E100*, W178*, and G231* may compete with wild-type LATS proteins for the binding partners of LCD1. Interestingly, LATS2-S83 within CNM is phosphorylated by Aurora-A to regulate the centrosomal localization and mitotic activity of LATS2 (Visser and Yang, 2010). Finally, the conserved residues in LCD1 could be critical for function. Mutations were found at certain conserved sites in LCD1 which include LATS1/2-R28/R16, S45/S33, L78/L77, and I81/I80. Moreover, N463S, P468S, H475Y, A483T, P493S, R502C, P506R/L, and W519C in LCD2 for LATS1 are predicted to be damaging. P468S is close to the phosphorylation site LATS1-S464 by NUAK, which promotes LATS1 degradation. Additionally, CDC2 phosphorylates S613 of LATS1. CDC2 forms a complex with LATS1 in the centrosome and phosphorylation of S613 occurs during mitosis (Visser and Yang, 2010). Mutations near S613, such as K607N and I615T, may interfere with phosphorylation at this site. Four lesser deleterious LATS2 mutations were found in LCD2, which contains the phosphorylation sites S408 and S446 by Chk1/2 in response to UV damage (Okada et al., 2011). Next to LCD2, LATS2-S380 is phosphorylated by Aurora A during mitosis, which is critical for Aurora A-LATS-Aurora B axis to regulate mitotic progression (Yabuta et al., 2011). Moreover, LATS2 S380 is located within an Ajuba-binding region of LATS2 (aa 376–396), which regulates the spindle apparatus formation (Visser and Yang, 2010). Mutations like R391H may affect the interactions and cell cycle control.

## Proline-rich region

LATS1 has a unique proline-rich region (Fig. 1A). Previous studies detected phosphorylation of T246 and T255 in P-stretch, as well as S336 located downstream (Hornbeck et al., 2012). T255A/N and S336G mutations in LATS1 would prevent phosphorylation of these residues, and the R252I/G mutations nearby may affect these phosphorylation events. Moreover, Y277 and S278 located within the LIMK-binding site of LATS1 were also found to be phosphorylated (Visser and Yang, 2010). While the functional significance of these phosphorylations is unknown, Y277C and S278C mutations clearly prevent phosphorylation of these two residues.

## Binding with YAP and KIBRA

The interaction between the Proline-Proline-x-Tyrosine (PPxY) motif and the hydrophobic pocket of WW domain is critical for the LATS1/2 binding with either their substrate YAP or their activator KIBRA. It has been reported that LATS1-Y559F and Y376A abrogated the binding with YAP (Visser and Yang, 2010), while LATS2-Y518A partially abolish the binding with KIBRA (Xiao et al., 2011). These interactions may be affected by the third proline mutation P375S in the first PPxY motif of LATS1 and the second proline mutation P516L in the only PPxY motif of LATS2.

## Binding with MOB, AJUBA and AMOTL2

The N-terminal regulatory domain (NTR) adjacent to the kinase domain is required for LATS1/2 activation by MOB, AJUBA, and AMOTL2 (Visser and Yang, 2010; Paramasivam et al., 2011; Xiao et al., 2011). Positively charged residues such as LATS1-R657/LATS2-K620, R660/R623, R682/R645, R694/R657, and R697/R660 in LATS1/2 NTR are conserved in the NDR subfamily from yeast to human. Previous studies have shown that LATS1 mutations (e.g. R657A, R660A and R694A) abolish their interaction with the negatively charged surface of MOB1A/B and kinase activity (Hergovich et al., 2006). Therefore, LATS1 mutants (R657C, R694C and R697G) and LATS2 mutants (R623W and R645L), are expected to become inactive in human cancer due to loss of interactions with LATS activators such as MOB. The *Drosophila* Wts-R702 residue is equivalent to LATS1/2-R694/R657 and critical for Mob-binding, kinase activity, and inhibition of tissue growth in development (Ho et al., 2010).

## The kinase domain

LATS1 and LATS2 are members of the AGC (named after PKA, PKG, and PKC) protein kinase family. Although crystal structures of some AGC proteins have been deciphered, there is no structure information available for LATS1/2 kinase domains. To estimate mutation-induced structural changes, we performed structure remodeling for LATS1/2 based on two AGC family proteins, ROCK and PKC. Conserved in most eukaryotic protein kinases, the N-terminal catalytic domain of LATS1/2 interacts with the phosphate donor ATP through a crucial network, composited by GxGxxGxV loop (LATS1: 712–719/ LATS2: 675–682), K734/697, E753/716, DxKxxN (828–833/791–795) and DFG motif (846–848/809–811) (“x” represents any aa) (Endicott et al., 2012; Hanks and Hunter, 1995). In addition, previous studies also have verified that mutants LATS1-K734M/LATS2-K697A/mLats2-K655M are all kinase-dead (Zhao et al., 2007; Wei et al., 2007; Visser and Yang, 2010). Mutated within or close to these conserved catalytic elements, LATS1 V719I/A, R744Q/L, A748T, R827T, R837H, L844M and LATS2 G675W, A678S, V682L, L693M, L699V, D800Y, G803C are most likely to disrupt ATP binding and catalysis. LATS1-R995C/L/H and LATS2-G909R also change highly conserved residues. Therefore, these cancer mutants may affect their kinase activity. Moreover, conserved residues in the kinase domain of LATS1/2 are expected to be important for their kinase activity. Mutations occurring in these residues included LATS1-N762/LATS2-N725, R806/R769, A810/A773, R827/R790, D837/D800, R854/R817, and R995/R958 (Fig. 1).

Unlike other AGC family members, LATS1/2 and the NDR subfamily members have an insert between the kinase subdomain VII and the activation segment (AS) in the subdomain VIII. Basic residues in the insert inhibit the activity of AS likely through an auto-inhibitory mechanism (Hergovich et al., 2006). Missense mutations in this region (e.g. LATS1-D871Y) may have an impact on the kinase activity (Fig. 1).

## Phosphorylation and activation by MST1/2 kinases

The phospho-S909/S872 and other residues in AS organize interaction between ATP and substrates of LATS1/2. Previous study has shown that LATS1 mutant S909A/D/E cannot be activated by the MST2 kinase. Therefore, LATS2-S872L would abolish the kinase activity, and LATS1-T913I and LATS2-T876N, A881V also likely damage catalytic activity. In the C-terminal of kinase IX and XI subdomains, the LATS2-G909 and LATS1-R995 are highly conserved among eukaryotic kinases (Endicott et al., 2012). LATS1-R995C/L/H and LATS2-G909R would probably damage kinase activity. LATS2-G909R and C953* has been experimentally shown to be defective for kinase activity and YAP regulation (Yu et al., 2013).

In the C-terminal domain (CTD) of AGC family kinase, the conserved NFD (Asn-Phe-Asp) motif interacts with hydrophobic pocket in the N-terminus of kinase domain to facilitate kinase activation (e.g. PKC, Leonard et al., 2011). In LATS1 NFD, N1038H may disrupt the kinase activity. In addition, LATS1-R1020T, P1028A/T, S1023C and LATS2-P996L, D998G may also affect this activation. LATS1/2 activation also requires phosphorylation of T1079/T1041 by MST1/2 in hydrophobic motif (HM), which has a consensus sequence F-x-x-Y/F-T-Y/F-K/R in the NDR protein subfamily (Hergovich et al., 2006). Thus, LATS2-T1041I/P mutations clearly abolish phosphorylation at this site to cause kinase inactivation. LATS1-R1082K, D1086Y and LATS2-E1039K, R1043L, D1048N may disrupt kinase activity as well. Mutations at conserved sites between LATS1/2 such as LATS1-F1015/LATS2-F978, R1020/R983, F1039/F1001, and D1086/D1048 may affect function of the C-terminus.

## Concluding remarks

Genetic analysis of Lats/Wts family genes using *Drosophila* and mice models has revealed their role as negative growth regulators and tumor suppressors in animal (Visser and Yang, 2010; Harvey et al., 2013; Yu and Guan, 2013). The fact that human LATS1 can functionally replace Wts in *Drosophila* supports that LATS may function as a tumor suppressor in human cells. From human cancer genome projects, an increasing number of mutations in *LATS1*/*2* are detected. Compared to some well-established cancer genes such as *TP53* and *Rb*, *LATS1* and *LATS2* are not frequently mutated. However, our *in silico* analysis provides supporting evidence that *LATS1*/*2* mutations drive human tumor development based on the following observations: 1) Cancer mutations in *hLATS1*/*2* do not appear to be random mutations. Damaging mutations have been accumulated more preferentially in important protein domains such as the kinase domain. Majority of the mutations including nonsense and frame-shift mutations clearly disrupt LATS1/2 function; 2) Some mutations occurred in regions and residues important for LATS1/2 activation. Examples include MST1/2 phosphorylation sites, MOB-binding domain, and the region critical for NF2 interaction; 3) In certain cancer types like stomach adenocarcinoma and uterine corpus endometrial carcinoma, mutation rates can be reasonably high (5.2%–5.9%). On the other hand, mechanisms other than gene mutation could also be effective to alter gene activity. In this regard, *LATS1*/*2* genes are known to be down-regulated by promoter methylation. Mutations in other Hippo pathway genes such as *MST1*/*2* are also expected to reduce *LATS1*/*2* function. While the functional significance of some of these mutations have been experimentally tested *in vivo* (Yu et al., 2013), results reported here further support that *LATS1*/*2* act normally as tumor suppressors and loss of their functions contributes to human cancer development.

## Electronic supplementary material

Below is the link to the electronic supplementary material.

Supplementary material 1 (PDF 462 kb)

Supplementary material 2 (PDF 348 kb)

Supplementary material 3 (PDF 358 kb)

Supplementary material 4 (PDF 326 kb)

Supplementary material 5 (PDF 34 kb)

## Abbreviations

CTD: C-terminal domain
HM: hydrophobic motif
LCD1: LATS conserved domain 1
LCD2: LATS conserved domain 2
NTR: N-terminal regulatory domain
PPxY: proline-proline-x-tyrosine
