# Supplementary material for: Mutation analysis of large tumor suppressor genes LATS1 and LATS2 supports a tumor suppressor role in human cancer
Source: Protein Cell. 2014 Dec 9;6(1):6–11. doi: 10.1007/s13238-014-0122-4 (PMC4286129; doi:10.1007/s13238-014-0122-4)
Supplement: Supplementary file 1 — Supplementary material 1 (PDF 462 kb) [file 13238_2014_122_MOESM1_ESM.pdf]

Cancer Study	Type	AA change	AA position	Cosmic	FIS
TCGA-CD-A4MI	Stomach(TCG.	A161V	161	Missense	diploid
HEC1B_ENDOMETRIUM	CCLE(Broad)	A47S	47	Missense	hetloss
HEC1A_ENDOMETRIUM	CCLE(Broad)	A47S	47	Missense	diploid
TCGA-CD-A4MJ	Stomach(TCG.	A483T	483	Missense	diploid
TCGA-CG-5733	Stomach(TCG.	A549S	549	Missense	diploid
TCGA-24-1419	Ovarian(TCGA	A748T	748	Missense	diploid
TCGA-24-1419	Ovarian(TCGA	A748T	748	Missense	diploid
TCGA-AA-3516	Colorectal(TC	A805V	805	Missense	diploid
TCGA-AA-3516	Colorectal(TC	A805V	805	Missense	diploid
SNU1040_LARGE_IN...	CCLE(Broad)	A810S	810	Missense	diploid
TCGA-D1-A161	Uterine(TCGA	A899fs	899	FSdel	diploid
TCGA-D1-A161	Uterine(TCGA	A899fs	899	FSdel	diploid
MCF7	NCI-60	D1043N	1043	Missense	hetloss
TCGA-HU-A4H5	Stomach(TCG.	D1086Y	1086	Missense	gain
TCGA-BB-4227	Head&neck(T	D837H	837	Missense	diploid
TCGA-BS-A0UV	Uterine(TCGA	D871Y	871	Missense	diploid
TCGA-BS-A0UV	Uterine(TCGA	D871Y	871	Missense	diploid
TCGA-66-2783	Lungsqu(TCG/	D994N	994	Missense	hetloss
TCGA-66-2783	Lungsqu(TCG/	D994N	994	Missense	hetloss
TCGA-B5-A0JY	Uterine(TCGA	E100*	100	Nonsense	diploid
TCGA-B5-A0JY	Uterine(TCGA	E100*	100	Nonsense	diploid
TCGA-C8-A26V	Breast(TCGA)	E36D	36	Missense	diploid
TCGA-05-4396	Lungadeno(T	E574*	574	Nonsense	hetloss
SKMES1_LUNG	CCLE(Broad)	E574*	574	Nonsense	gain
KMS11_HAEMATOPOI...	CCLE(Broad)	E594K	594	Missense	diploid
LC1F_LUNG	CCLE(Broad)	E606G	606	Missense	hetloss
LC1SQSF_LUNG	CCLE(Broad)	E606G	606	Missense	hetloss
HT115_LARGE_INTE...	CCLE(Broad)	E689*	689	Nonsense	diploid
647V_URINARY_TRACT	CCLE(Broad)	E802K	802	Missense	diploid
TCGA-D8-A147	Breast(TCGAp	E920fs	920	FSdel	hetloss
HCC1569_BREAST	CCLE(Broad)	F1010fs	1010	FSdel	hetloss
TCGA-BR-8680	Stomach(TCG.	F1015L	1015	Missense	hetloss
HCT116_LARGE_INT...	CCLE(Broad)	F532fs	532	FSdel	diploid
TCGA-AA-A00N	Colorectal(TC	F641L	641	Missense	diploid
TCGA-AA-A00N	Colorectal(TC	F641L	641	Missense	diploid
TCGA-BS-A0UF	Uterine(TCGA	F641L	641	Missense	diploid
TCGA-BS-A0UF	Uterine(TCGA	F641L	641	Missense	diploid
SW403_LARGE_INTE...	CCLE(Broad)	G1106A	1106	Missense	gain
B109	Bladder(BGI)	G113E	113	Missense	NA
TCGA-CQ-5324	Head&neck(T	G166_splice	166	Splice	diploid

TCGA-CQ-5324	Head&neck(T	G166_splice	166	Splice	diploid
H090284	Liver(LGGM)	G231*	231	Nonsense	diploid
H090284	Liver(LGGM)	G448W	448	Missense	diploid
TCGA-DR-A0ZM	Cervical(TCGA	G535E	535	Missense	diploid
TCGA-BP-4989	ccRCC(TCGAp	G554E	554	Missense	diploid
TCGA-BP-4989	ccRCC(TCGA)	G554E	554	Missense	diploid
TCGA-BR-8384	Stomach(TCG.	G787A	787	Missense	diploid
TCGA-55-7907	Lungadeno(T	G787V	787	Missense	diploid
BECKER_CENTRAL_N...	CCLE(Broad)	G823V	823	Missense	diploid
H110061	Liver(LGGM)	H359L	359	Missense	diploid
TCGA-91-6840	Lungadeno(T	H417D	417	Missense	hetloss
MEL-JWCI-WGS-22	Melanoma(Br	H475Y	475	Missense	NA
HEC151_ENDOMETRIUM	CCLE(Broad)	H52Y	52	Missense	diploid
GP2D_LARGE_INTES...	CCLE(Broad)	I131M	131	Missense	diploid
NCIH1930_LUNG	CCLE(Broad)	I131V	131	Missense	hetloss
KS1_CENTRAL_NERV...	CCLE(Broad)	I220V	220	Missense	hetloss
TCGA-CF-A1HR	Bladder(TCGA	I288M	288	Missense	hetloss
TCGA-CF-A1HR	Bladder(TCGA	I288M	288	Missense	hetloss
TGBC11TKB_STOMACH	CCLE(Broad)	I81M	81	Missense	diploid
NCIH1573_LUNG	CCLE(Broad)	K1005*	1005	Nonsense	hetloss
NCIH1573_LUNG	CCLE(Broad)	K1005N	1005	Missense	hetloss
TCGA-AP-A0LM	Uterine(TCGA	K607N	607	Missense	diploid
TCGA-AP-A0LM	Uterine(TCGA	K607N	607	Missense	diploid
KM12_LARGE_INTES...	CCLE(Broad)	L109S	109	Missense	diploid
KM12	NCI-60	L109S	109	Missense	diploid
TCGA-30-1891	Ovarian(TCGA	L78fs	78f	FSdel	diploid
TCGA-30-1891	Ovarian(TCGA	L78fs	78f	FSdel	diploid
NCIH1836_LUNG	CCLE(Broad)	L793Q	793	Missense	hetloss
GCT_SOFT_TISSUE	CCLE(Broad)	M310V	310	Missense	gain
TCGA-A6-2676	Colorectal(TC	M704V	704	Missense	gain
TCGA-A6-2676	Colorectal(TC	M704V	704	Missense	gain
P08-716	Prostate(Broa	M704V	704	Missense	diploid
YUDAB	Melanoma(Ya	M782I	782	Missense	NA
TCGA-AA-3681	Colorectal(TC	M790T	790	Missense	diploid
TCGA-AA-3681	Colorectal(TC	M790T	790	Missense	diploid
TCGA-BR-8680	Stomach(TCG.	N1038H	1038	Missense	hetloss
TCGA-55-7281	Lungadeno(T	N463S	463	Missense	diploid
TCGA-05-4424	Lungadeno(T	N471S	471	Missense	diploid
TCGA-05-4424	Lungadeno(T	N471S	471	Missense	diploid

TCGA-BR-4361	Stomach(TCG.	N551S	551	Missense	diploid
TCGA-AN-A046	Breast(TCGA)	N999D	999	Missense	diploid
TCGA-A8-A07R	Breast(TCGAp	P1028A	1028	Missense	diploid
TCGA-A8-A07R	Breast(TCGA)	P1028A	1028	Missense	hetloss
TCGA-13-0714	Ovarian(TCGA	P1028T	1028	Missense	hetloss
LUAD-CHTN-MAD06-...	Lungadeno(Br	P158S	158	Missense	diploid
TCGA-HT-8564	Glioma(TCGA)	P237fs	237	FSdel	hetloss
SNUC5_LARGE_INTE...	CCLE(Broad)	P237fs	237	FSdel	diploid
KMS12BM_HAEMATOP...	CCLE(Broad)	P237Q	237	Missense	diploid
RERFGC1B_STOMACH	CCLE(Broad)	P237Q	237	Missense	diploid
MOTN1_HAEMATOPOI...	CCLE(Broad)	P237Q	237	Missense	diploid
SNU119_OVARY	CCLE(Broad)	P237Q	237	Missense	HOMDEL
SNU620_STOMACH	CCLE(Broad)	P237Q	237	Missense	diploid
HMC18_BREAST	CCLE(Broad)	P237Q	237	Missense	hetloss
JHOS2_OVARY	CCLE(Broad)	P237Q	237	Missense	hetloss
IALM_LUNG	CCLE(Broad)	P237Q	237	Missense	diploid
TF1_HAEMATOPOIET...	CCLE(Broad)	P237Q	237	Missense	gain
RERFLCAD1_LUNG	CCLE(Broad)	P237Q	237	Missense	hetloss
LC1F_LUNG	CCLE(Broad)	P237Q	237	Missense	hetloss
NCIH2227_LUNG	CCLE(Broad)	P250S	250	Missense	hetloss
MEWO_SKIN	CCLE(Broad)	P258S	258	Missense	diploid
LUAD-RT-S01702	Lungadeno(Br	P266fs	266	FSdel	diploid
TCGA-78-7155	Lungadeno(TC	P292fs	292	FSdel	diploid
TCGA-D3-A51R	Melanoma(TC	P292L	292	Missense	diploid
TCGA-AA-3984	Colorectal(TC	P301H	301	Missense	gain
TCGA-AA-3984	Colorectal(TC	P301H	301	Missense	AMP
TCGA-EE-A29D	Melanoma(TC	P301S	301	Missense	hetloss
TCGA-BA-6869	Head&neck(T	P375S	375	Missense	diploid
TCGA-BA-6869	Head&neck(T	P375S	375	Missense	diploid
KYSE150_OESOPHAGUS	CCLE(Broad)	P377S	377	Missense	diploid
EVSAT_BREAST	CCLE(Broad)	P434R	434	Missense	gain
TCGA-BR-A4QL	Stomach(TCG.	P445L	445	Missense	diploid
SW1271_LUNG	CCLE(Broad)	P452H	452	Missense	diploid
ME009	Melanoma(Br	P468S	468	Missense	NA
TCGA-ER-A42L	Melanoma(TC	P493S	493	Missense	hetloss
B74	Bladder(BGI)	P506L	506	Missense	NA
TCGA-13-1483	Ovarian(TCGA	P506R	506	Missense	hetloss
TCGA-13-1483	Ovarian(TCGA	P506R	506	Missense	hetloss
HCT_116	NCI-60	P531fs	531	FSdel	diploid
TCGA-EE-A29L	Melanoma(TC	P568L	568	Missense	hetloss
TCGA-AX-A063	Uterine(TCGA	P579S	579	Missense	diploid

TCGA-AX-A063	Uterine(TCGA	P579S	579	Missense	diploid
LNCAPCLONEFGC_PR...	CCLE(Broad)	Q188R	188	Missense	hetloss
TCGA-BR-6452	Stomach(TCG.	Q678H	678	Missense	diploid
TCGA-CF-A3MG	Bladder(TCGA	Q863E	863	Missense	hetloss
MM-0508	MM(Broad)	Q903*	903	Nonsense	NA
TCGA-04-1331	Ovarian(TCGA	R1020T	1020	Missense	hetloss
TCGA-04-1331	Ovarian(TCGA	R1020T	1020	Missense	hetloss
TCGA-BR-4280	Stomach(TCG.	R1082K	1082	Missense	diploid
TCGA-IB-7651	Panceas(TCGA	R1125H	1125	Missense	diploid
RS411_HAEMATOPOI...	CCLE(Broad)	R1125H	1125	Missense	diploid
NCCSTCK140_STOMACH	CCLE(Broad)	R174C	174	Missense	gain
HEC151_ENDOMETRIUM	CCLE(Broad)	R174C	174	Missense	diploid
TCGA-AX-A0J0	Uterine(TCGA	R233S	233	Missense	diploid
TCGA-AX-A0J0	Uterine(TCGA	R233S	233	Missense	diploid
16913	Lungadeno(TS	R252I	252	Missense	NA
TCGA-EE-A3JE	Melanoma(TC	R287*	287	Nonsense	diploid
TCGA-B0-5710	ccRCC(TCGAp	R28Q	28	Missense	diploid
TCGA-B0-5710	ccRCC(TCGA)	R28Q	28	Missense	diploid
TCGA-05-4396	Lungadeno(TC	R35L	35	Missense	hetloss
JHUEM7_ENDOMETRIUM	CCLE(Broad)	R35W	35	Missense	diploid
TCGA-BT-A3PJ	Bladder(TCGA	R35W	35	Missense	diploid
16660	Lungadeno(TS	R502C	502	Missense	NA
BT474_BREAST	CCLE(Broad)	R63Q	63	Missense	hetloss
TCGA-A5-A0G9	Uterine(TCGA	R657C	657	Missense	diploid
TCGA-A5-A0G9	Uterine(TCGA	R657C	657	Missense	diploid
TCGA-24-1603	Ovarian(TCGA	R694C	694	Missense	hetloss
TCGA-24-1603	Ovarian(TCGA	R694C	694	Missense	hetloss
TCGA-B9-5156	pRCC(TCGA)	R697G	697	Missense	diploid
TCGA-AG-A002	Colorectal(TC	R737*	737	Nonsense	diploid
TCGA-AG-A002	Colorectal(TC	R737*	737	Nonsense	diploid
ISHIKAWAHERAKLIO...	CCLE(Broad)	R737*	737	Nonsense	diploid
TCGA-AX-A0J0	Uterine(TCGA	R737*	737	Nonsense	diploid
TCGA-AX-A0J0	Uterine(TCGA	R737*	737	Nonsense	diploid
TCGA-E2-A14O	Breast(TCGAp	R737*	737	Nonsense	diploid
HEC251_ENDOMETRIUM	CCLE(Broad)	R744*	744	Nonsense	diploid
SNU81_LARGE_INTE...	CCLE(Broad)	R744*	744	Nonsense	diploid
TCGA-AX-A05Z	Uterine(TCGA	R744*	744	Nonsense	diploid
TCGA-AX-A05Z	Uterine(TCGA	R744*	744	Nonsense	diploid
CL40_LARGE_INTES...	CCLE(Broad)	R744*	744	Nonsense	diploid
TCGA-75-5147	Lungadeno(TC	R744L	744	Missense	diploid

TCGA-AA-A00N	Colorectal(TC(	R744Q	744	Missense	diploid
TCGA-AA-A00N	Colorectal(TC(	R744Q	744	Missense	diploid
TCGA-D6-6517	Head&neck(T(	R767L	767	Missense	diploid
TCGA-D6-6517	Head&neck(T(	R767L	767	Missense	diploid
TCGA-B5-A0JY	Uterine(TCGA	R82*	82	Nonsense	diploid
TCGA-B5-A0JY	Uterine(TCGA	R82*	82	Nonsense	diploid
HEC251_ENDOMETRIUM	CCLE(Broad)	R82*	82	Nonsense	diploid
YURIDA	Melanoma(Ya	R82*	82	Nonsense	NA
TCGA-BS-A0UV	Uterine(TCGA	R82*	82	Nonsense	diploid
TCGA-BS-A0UV	Uterine(TCGA	R82*	82	Nonsense	diploid
LUAD-CHTN-3090416	Lungadeno(Br	R827T	827	Missense	diploid
TCGA-F1-6177	Stomach(TCG.	R82Q	82	Missense	diploid
ESO-2143	Esophagus(Br	R82Q	82	Missense	NA
TCGA-AC-A23H	Breast(TCGA)	R838G	838	Missense	diploid
TCGA-22-4604	Lungsqu(TCG/	R854K	854	Missense	diploid
TCGA-22-4604	Lungsqu(TCG/	R854K	854	Missense	diploid
TCGA-05-4396	Lungadeno(TC	R96L	96	Missense	hetloss
TCGA-EE-A20C	Melanoma(TC	R96Q	96	Missense	diploid
TCGA-AA-A02H	Colorectal(TC(	R990Q	990	Missense	hetloss
TCGA-AA-A02H	Colorectal(TC(	R990Q	990	Missense	hetloss
SKHEP1_LIVER	CCLE(Broad)	R995H	995	Missense	hetloss
LOXIMVI_SKIN	CCLE(Broad)	R995L	995	Missense	diploid
LOXIMVI	NCI-60	R995L	995	Missense	diploid
TCGA-BB-4227	Head&neck(T(	S1023C	1023	Missense	diploid
NCIH2110_LUNG	CCLE(Broad)	S278C	278	Missense	hetloss
YUAKER	Melanoma(Ya	S308F	308	Missense	NA
TCGA-A6-2676	Colorectal(TC(	S336G	336	Missense	gain
TCGA-A6-2676	Colorectal(TC(	S336G	336	Missense	gain
TCGA-CF-A1HR	Bladder(TCGA	S387F	387	Missense	hetloss
TCGA-CF-A1HR	Bladder(TCGA	S387F	387	Missense	hetloss
MEL-Ma-Mel-102	Melanoma(Br	S438F	438	Missense	NA
TCGA-CG-5721	Stomach(TCG.	S444P	444	Missense	diploid
TCGA-AX-A0J1	Uterine(TCGA	S45Y	45	Missense	diploid
TCGA-AX-A0J1	Uterine(TCGA	S45Y	45	Missense	diploid
TCGA-91-6829	Lungadeno(TC	S803T	803	Missense	diploid
TCGA-91-6829	Lungadeno(TC	S803T	803	Missense	diploid
16678	Lungadeno(TS	T255N	255	Missense	NA
TCGA-FP-7829	Stomach(TCG.	T367I	367	Missense	diploid
HCT_15	NCI-60	T851I	851	Missense	diploid
HCT15_LARGE_INTE...	CCLE(Broad)	T851I	851	Missense	diploid
HRT18_LARGE_INTE...	CCLE(Broad)	T851I	851	Missense	diploid

TCGA-AA-A00N	Colorectal(TC	V1057A	1057	Missense	diploid
TCGA-AA-A00N	Colorectal(TC	V1057A	1057	Missense	diploid
S00827	LungSC(CLCGI	V234L	234	Missense	NA
TCGA-22-4613	Lungsqu(TCG/	V25F	25	Missense	diploid
TCGA-22-4613	Lungsqu(TCG/	V25F	25	Missense	diploid
HS729_SOFT_TISSUE	CCLE(Broad)	V284I	284	Missense	gain
TCGA-GD-A3OP	Bladder(TCGA	W178*	178	Nonsense	hetloss
TCGA-GF-A3OT	Melanoma(TC	W268*	268	Nonsense	diploid
TCGA-44-7670	Lungadeno(T	W519C	519	Missense	hetloss
S00050	LungSC(CLCGI	Y200S	200	Missense	NA
HEC151_ENDOMETRIUM	CCLE(Broad)	Y862C	862	Missense	diploid

3D	VS	Allele Frequen	#Mut in Sampl	SIFT	PROVEAN	PolyPhen-2
U	Low	0.15	758	Damaging	Neutral	possiblydamag
U	Neutral	0.96	324	Tolerated	Neutral	benign
U	Neutral	0.49	365	Tolerated	Neutral	benign
U	Low	0.22	530	Damaging	Neutral	possiblydamag
U	Low	0.27	882	Tolerated	Neutral	benign
V	Neutral	NA	34	Damaging	Deleterious	neutral
V	Neutral	NA	34	Damaging	Deleterious	neutral
V	Neutral	NA	693	Damaging	Deleterious	neutral
U	Neutral	NA	681	Damaging	Deleterious	neutral
U	Low	0.42	1308	Damaging	Deleterious	probablydama
U		NA	70			
U		NA	70			
U	Medium	NA	384	Damaging	Deleterious	probablydamag
U	Medium	0.1	229	Damaging	Deleterious	probablydamag
U	Low	0.37	213	Damaging	Deleterious	probablydama
U	Low	0.16	7801	Damaging	Deleterious	probably/poss
U	Low	0.16	7553	Damaging	Deleterious	probably/poss
U	Neutral	0.02	228	Tolerated	Neutral	neutral
U	Neutral	0.02	214	Tolerated	Neutral	neutral
U		0.22	8620			
U		0.22	8890			
U	Medium	NA	53	Damaging	Neutral	medium
U		NA	9220			
U		0.28	47			
U	Low	0.72	55	Tolerated	Neutral	benign
U	Low	0.51	58	Tolerated	Neutral	benign
U	Low	0.97	56	Tolerated	Neutral	benign
U		0.41	640			
U	Medium	0.46	65	Damaging	Deleterious	possiblydamag
U		NA	108			
U		0.51	161			
U	Medium	0.53	5317	Damaging	Deleterious	benign
U		0.48	278			
V	Medium	NA	3513	Tolerated	Deleterious	benign
V	Medium	NA	3492	Tolerated	Deleterious	benign
U	Medium	0.32	7391	Tolerated	Deleterious	benign
U	Medium	0.32	7176	Tolerated	Deleterious	benign
U	Neutral	0.22	78	Damaging	Neutral	benign
U	Neutral	NA	207	Damaging	Deleterious	probablydamag
U		0.35	66			

U			0.35	69			
U			0.23	935			
U	Low		0.06	935	Damaging	Deleterious	probablydamag
U	Low		0.17	1312	Tolerated	Neutral	benign
V	Low	NA		66	Damaging	Neutral	low
V	Low	NA		58	Damaging	Neutral	low
U	Medium		0.1	40	Damaging	Deleterious	probablydamag
U	Medium		0.21	981	Damaging	Deleterious	medium
U	Medium		0.29	32	Damaging	Deleterious	probablydama
U	Neutral		0.1	134	Tolerated	Neutral	possiblydamag
U	Low		0.21	108	Tolerated	Neutral	low
U	Low	NA		935	Damaging	Neutral	possiblydamag
U	Neutral		0.44	381	Tolerated	Neutral	benign
U	Neutral		0.55	578	Damaging	Neutral	benign
U	Neutral		0.55	114	Tolerated	Neutral	benign
U	Neutral		0.31	56	Tolerated	Neutral	benign
U	Neutral		0.1	278	Tolerated	Neutral	neutral
U	Neutral		0.1	290	Tolerated	Neutral	neutral
U	Medium		0.13	217	Damaging	Neutral	probablydama
U			0.99	175			
U	Medium		1	175	Damaging	Deleterious	probablydama
U	Medium		0.22	10148	Damaging	Neutral	benign
U	Medium		0.22	10489	Damaging	Neutral	benign
U	Neutral		0.53	326	Damaging	Deleterious	benign
U	Neutral	NA		1774	Damaging	Deleterious	benign
U		NA		52			
U		NA		52			
U	Low		0.32	61	Damaging	Deleterious	probablydama
U	Neutral		0.11	86	Tolerated	Neutral	benign
V	Neutral	NA		595	Damaging	Deleterious	neutral
U	Neutral	NA		598	Damaging	Deleterious	neutral
U	Neutral	NA		36	Damaging	Deleterious	neutral
U	Low	NA		590	Damaging	Deleterious	probablydamag
V	Low	NA		94	Damaging	Deleterious	low
V	Low	NA		95	Damaging	Deleterious	low
U	Low		0.2	5317	Damaging	Deleterious	benign
U	Low		0.41	413	Tolerated	Neutral	low
U	Neutral		0.22	698	Tolerated	Neutral	benign
U	Neutral	NA		733	Tolerated	Neutral	benign

U	Low		0.04	2314	Tolerated	Neutral	benign
U	Neutral	NA		4185	Tolerated	Deleterious	benign
U	Medium	NA		174	Damaging	Deleterious	medium
U	Medium	NA		346	Damaging	Deleterious	medium
V	High	NA		60	Damaging	Deleterious	high
U	Low	NA		1834	Damaging	Neutral	low
U			0.11	432			
U			0.45	363			
U	Low		0.57	40	Damaging	Neutral	probablydama
U	Low		0.33	42	Damaging	Neutral	probablydama
U	Low		0.51	39	Damaging	Neutral	probablydama
U	Low		1	42	Damaging	Neutral	probablydama
U	Low		0.75	42	Damaging	Neutral	probablydama
U	Low		0.4	48	Damaging	Neutral	probablydama
U	Low		1	40	Damaging	Neutral	probablydama
U	Low		1	60	Damaging	Neutral	probablydama
U	Low		0.34	54	Damaging	Neutral	probablydama
U	Low		0.67	75	Damaging	Neutral	probablydama
U	Low		0.57	58	Damaging	Neutral	probablydama
U	Low		1	57	Tolerated	Neutral	benign
U	Low		0.49	507	Tolerated	Neutral	possiblydamag
U		NA		478			
U			0.81	1367			
U	Low		0.44	554	Damaging	Deleterious	probablydama
V	Low	NA		3184	Damaging	Neutral	low
V	Low	NA		3197	Damaging	Neutral	low
U	Low		0.53	2906	Tolerated	Neutral	possiblydamag
U	Low		0.57	407	Tolerated	Neutral	probably/poss
U	Low		0.57	418	Tolerated	Neutral	probably/poss
U	Neutral		0.67	80	Tolerated	Neutral	benign
U	Low		0.24	72	Damaging	Neutral	benign
U	Low		0.37	1534	Damaging	Deleterious	possiblydamag
U	Low		0.38	138	Damaging	Neutral	probably/poss
U	Low	NA		2151	Damaging	Neutral	benign
U	Medium		0.06	788	Damaging	Deleterious	possiblydamag
U	Medium	NA		103	Damaging	Deleterious	probablydamag
V	Medium	NA		45	Damaging	Deleterious	probablydamag
V	Medium	NA		44	Damaging	Deleterious	probablydamag
U		NA		1602			
U	Low		0.45	1569	Tolerated	Neutral	benign
U	Neutral		0.37	685	Tolerated	Neutral	benign

U	Neutral		0.37	709	Tolerated	Neutral	benign
U	Low		0.58	360	Damaging	Neutral	probably/poss
U	Medium		0.06	6281	Damaging	Deleterious	possiblydamag
U	Neutral		0.42	131	Tolerated	Deleterious	neutral
U		NA		77			
V	Medium	NA		78	Damaging	Deleterious	medium
V	Medium	NA		75	Damaging	Deleterious	medium
U	Medium		0.33	646	Damaging	Deleterious	probablydama
U	Neutral		0.16	13534	Damaging	Neutral	benign
U	Neutral		0.5	115	Damaging	Neutral	benign
U	Low		0.39	45	Damaging	Deleterious	probably/poss
U	Low		0.45	381	Damaging	Deleterious	probably/poss
U	Low		0.43	6645	Damaging	Neutral	probablydama
U	Low		0.43	6913	Damaging	Neutral	probablydama
U	Low	NA		5	Damaging	Neutral	low
U			0.06	232			
U	Neutral	NA		33	Tolerated	Neutral	neutral
U	Neutral	NA		32	Tolerated	Neutral	neutral
U	Medium	NA		9220	Damaging	Deleterious	probablydamag
U	Medium		0.47	825	Damaging	Deleterious	probablydama
U	Medium		0.17	430	Damaging	Deleterious	probablydama
U	Medium	NA		36	Damaging	Deleterious	medium
U	Low		0.36	109	Tolerated	Neutral	benign
U	Medium		0.11	199	Damaging	Deleterious	medium
U	Medium		0.11	196	Damaging	Deleterious	medium
V	Medium	NA		24	Damaging	Deleterious	medium
V	Medium	NA		24	Damaging	Deleterious	medium
U	Low		0.02	76	Damaging	Deleterious	probablydamag
V		NA		9896			
V		NA		9874			
U			0.51	314			
U			0.47	6913			
U			0.47	6645			
U		NA		33			
U			0.5	1171			
U			0.51	912			
U			0.23	5677			
U			0.23	5513			
U			0.98	40			
U	Neutral	NA		46	Damaging	Deleterious	neutral

V	Neutral	NA		3513	Damaging	Deleterious	probablydamag
V	Neutral	NA		3492	Damaging	Deleterious	probablydamag
U	Low		0.28	63	Damaging	Deleterious	probably/poss
U	Low		0.28	64	Damaging	Deleterious	probably/poss
U			0.35	8890			
U			0.35	8620			
U			0.48	1171			
U		NA		137			
U			0.34	7553			
U			0.34	7801			
U	Medium	NA		155	Damaging	Deleterious	medium
U	Medium		0.49	1083	Damaging	Neutral	possiblydamag
U	Medium		0.45	189	Damaging	Neutral	possiblydamag
U	Neutral	NA		3358	Tolerated	Deleterious	benign
U	Neutral		0.18	234	Tolerated	Deleterious	neutral
U	Neutral		0.18	230	Tolerated	Deleterious	neutral
U	Neutral	NA		9220	Tolerated	Neutral	benign
U	Neutral		0.34	1605	Tolerated	Neutral	benign
V	Neutral	NA		57	Damaging	Neutral	neutral
V	Neutral	NA		57	Damaging	Neutral	neutral
U	Medium		1	31	Damaging	Deleterious	probablydama
U	Medium		0.22	52	Damaging	Deleterious	probablydama
U	Medium	NA		296	Damaging	Deleterious	probablydama
U	Low		0.12	213	Damaging	Neutral	benign
U	Low		1	196	Damaging	Neutral	probablydama
U	Neutral	NA		1088	Damaging	Neutral	possiblydamag
V	Neutral	NA		595	Tolerated	Neutral	neutral
U	Neutral	NA		598	Tolerated	Neutral	neutral
U	Neutral		0.13	290	Damaging	Neutral	neutral
U	Neutral		0.13	278	Damaging	Neutral	neutral
U	Low	NA		1229	Damaging	Neutral	possiblydamag
U	Neutral		0.2	3370	Tolerated	Neutral	benign
U	Medium		0.42	3747	Damaging	Neutral	probablydama
U	Medium		0.42	3848	Damaging	Neutral	probablydama
U	Neutral		0.11	578	Tolerated	Neutral	neutral
U	Neutral	NA		596	Tolerated	Neutral	neutral
U	Low	NA		29	Tolerated	Neutral	low
U	Neutral		0.29	207	Damaging	Neutral	benign
U	Low	NA		4766	Damaging	Deleterious	probablydama
U	Low		0.47	741	Damaging	Deleterious	probablydama
U	Low		0.47	599	Damaging	Deleterious	probablydama

V	Neutral	NA		3513	Damaging	Neutral	neutral
V	Neutral	NA		3492	Damaging	Neutral	neutral
U	Low	NA		377	Tolerated	Neutral	low
U	Neutral		0.24	554	Damaging	Neutral	neutral
U	Neutral		0.24	567	Damaging	Neutral	neutral
U	Low		0.64	35	Tolerated	Neutral	benign
U			0.04	366			
U			0.28	421			
U	Medium		0.16	950	Damaging	Deleterious	medium
V	Low	NA		204	Damaging	Neutral	low
U	Low		0.57	381	Damaging	Deleterious	probablydama

MutationAss	SNV in dbSN	MAF(Minor Allel Frequency) in dbSNP
low		
neutral		
neutral		
low	A483T,G>A	
low		
ly/possiblydamaging		
ly/possiblydamaging		
obablydamaging		
obablydamaging		
low		
medium		
medium		
low		
low		
low		
benign		
benign		
benign		
low		
low		
low		
medium		
medium		
medium	F641L3C>A	
medium	F641L3C>A	
medium	F641L3C>A	
medium	F641L3C>A	
neutral	G1106A,G>C	
neutral		

low		
low		
obablydamaging		
obablydamaging		
medium		
obablydamaging		
medium		
neutral		
obablydamaging		
low		
neutral		
neutral		
neutral	I131V,1,A>G	0.0009
neutral		
benign		
benign		
medium		
medium		
medium		
medium		
neutral		
neutral		
low		
neutral		
blydamaging/b	M704V,A>G	
blydamaging/b	M704V,A>G	
blydamaging/b	M704V,A>G	
low		
obablydamaging		
obablydamaging		
low		
benign		
neutral		
neutral		

low		
neutral		
obablydamaging		
obablydamaging		
obablydamaging		
benign		
low	P237Q,C>A	0.0014
low	P237Q,C>A	0.0014
low	P237Q,C>A	0.0014
low	P237Q,C>A	0.0014
low	P237Q,C>A	0.0014
low	P237Q,C>A	0.0014
low	P237Q,C>A	0.0014
low	P237Q,C>A	0.0014
low	P237Q,C>A	0.0014
low	P237Q,C>A	0.0014
low	P237Q,C>A	0.0014
low		
low		
low		
›ly/possiblydamaging		
›ly/possiblydamaging		
low		
low		
low		
neutral		
low		
low		
low		
low		
low		
medium		
medium		
medium		
low		
neutral		

neutral		
low		
medium		
benign		
obablydamaging		
obablydamaging		
medium		
neutral		
neutral		
low	R174C,C>T	0.0005
low	R174C,C>T	0.0005
low		
low		
blydamaging/benign		
benign		
benign		
medium		
medium		
medium		
obablydamaging		
low		
obablydamaging		
obablydamaging		
obablydamaging		
obablydamaging		
low	R697G,A>C	
obablydamaging		

neutral	R744Q,G>A	0.0005
neutral	R744Q,G>A	0.0005
low		
low		
obablydamaging		
medium		
medium		
neutral		
gn/possiblydamaging		
gn/possiblydamaging		
neutral		
neutral	R96Q,G>A	
blydamaging/benign		
blydamaging/benign		
medium		
medium		
medium		
low		
low		
neutral		
benign		
benign		
benign		
benign		
low		
neutral		
medium		
medium		
benign		
benign		
benign		
neutral		
low		
low		
low		

benign
benign
obablydamaging
benign
benign
low

obablydamaging
ossiblydamaging
low
