# Supplementary material for: Mutation analysis of large tumor suppressor genes LATS1 and LATS2 supports a tumor suppressor role in human cancer
Source: Protein Cell. 2014 Dec 9;6(1):6–11. doi: 10.1007/s13238-014-0122-4 (PMC4286129; doi:10.1007/s13238-014-0122-4)
Supplement: Supplementary file 2 — Supplementary material 2 (PDF 348 kb) [file 13238_2014_122_MOESM2_ESM.pdf]

GeneName(/ represent overlapping mutations between tw	Transcript	SampleName
LATS1_ENST00000253339/ENST00000543571	ENST00000253339	TCGA-22-461
LATS1_ENST00000253339/ENST00000543571	ENST00000253339	TCGA-B0-571
LATS1_ENST00000253339/ENST00000543571	ENST00000253339	TCGA-C8-A26
LATS1_ENST00000253339/ENST00000543571	ENST00000253339	TCGA-AX-A0J
LATS1_ENST00000253339/ENST00000543571	ENST00000253339	HCC4T
LATS1_ENST00000253339/ENST00000543571	ENST00000253339	HX13T
LATS1_ENST00000253339/ENST00000543571	ENST00000253339	TCGA-AA-349
LATS1_ENST00000543571	ENST00000543571	TCGA-30-189
LATS1_ENST00000253339/ENST00000543571	ENST00000253339	TCGA-B5-A0J
LATS1_ENST00000253339/ENST00000543571	ENST00000253339	TCGA-BS-A0U
LATS1_ENST00000253339/ENST00000543571	ENST00000253339	YURIDA
LATS1_ENST00000253339/ENST00000543571	ENST00000253339	ESO-2143
LATS1_ENST00000253339/ENST00000543571	ENST00000253339	TCGA-B5-A0J
LATS1_ENST00000253339/ENST00000543571	ENST00000253339	LUAD-CHTN-
LATS1_ENST00000253339/ENST00000543571	ENST00000253339	TCGA-GD-A3
LATS1_ENST00000253339/ENST00000543571	ENST00000253339	4_RESISTANT
LATS1_ENST00000253339/ENST00000543571	ENST00000253339	S00050
LATS1_ENST00000253339/ENST00000543571	ENST00000253339	TCGA-AX-A0J
LATS1_ENST00000253339/ENST00000543571	ENST00000253339	S00827
LATS1_ENST00000253339/ENST00000543571	ENST00000253339	TCGA-AD-696
LATS1_ENST00000253339/ENST00000543571	ENST00000253339	TCGA-AD-688
LATS1	ENST00000543571	16913
LATS1_ENST00000253339/ENST00000543571	ENST00000253339	TCGA-G4-658
LATS1_ENST00000253339/ENST00000543571	ENST00000253339	TCGA-D5-653
LATS1	ENST00000543571	16678
LATS1_ENST00000543571	ENST00000543571	LAU165
LATS1_ENST00000253339/ENST00000543571	ENST00000253339	LUAD-RT-S01
LATS1_ENST00000253339/ENST00000543571	ENST00000253339	TCGA-CI-6624
LATS1_ENST00000253339/ENST00000543571	ENST00000253339	TCGA-CF-A1H
LATS1_ENST00000543571	ENST00000543571	TCGA-AA-398
LATS1_ENST00000253339/ENST00000543571	ENST00000253339	YUAKER
LATS1_ENST00000543571	ENST00000543571	SA106
LATS1_ENST00000543571	ENST00000543571	TCGA-A6-267
LATS1_ENST00000253339/ENST00000543571	ENST00000253339	TCGA-CF-A1H
LATS1_ENST00000253339	ENST00000253339	112241
LATS1_ENST00000253339	ENST00000253339	108422
LATS1_ENST00000253339/ENST00000543571	ENST00000253339	TCGA-91-684
LATS1_ENST00000253339/ENST00000543571	ENST00000253339	TCGA-80-561
LATS1_ENST00000253339/ENST00000543571	ENST00000253339	TCGA-55-728
LATS1_ENST00000253339/ENST00000543571	ENST00000253339	TCGA-05-442

LATS1_ENST00000253339/ENST00000543571	ENST00000253339	TCGA-G4-632[illegible]
LATS1_ENST00000253339/ENST00000543571	ENST00000253339	TCGA-CK-495[illegible]
LATS1	ENST00000543571	16660
LATS1_ENST00000253339	ENST00000253339	TCGA-13-148[illegible]
LATS1_ENST00000253339/ENST00000543571	ENST00000253339	TCGA-44-767[illegible]
LATS1_ENST00000253339/ENST00000543571	ENST00000253339	HCT-116
LATS1_ENST00000253339/ENST00000543571	ENST00000253339	TCGA-DR-A0Z[illegible]
LATS1_ENST00000253339/ENST00000543571	ENST00000253339	TCGA-BP-498[illegible]
LATS1_ENST00000253339/ENST00000543571	ENST00000253339	TCGA-AX-A06[illegible]
LATS1_ENST00000253339/ENST00000543571	ENST00000253339	ccRCC-10
LATS1_ENST00000253339/ENST00000543571	ENST00000253339	TCGA-AP-A0L[illegible]
LATS1_ENST00000253339/ENST00000543571	ENST00000253339	TCGA-A3-334[illegible]
LATS1_ENST00000253339/ENST00000543571	ENST00000253339	LC_S49
LATS1_ENST00000253339/ENST00000543571	ENST00000253339	TCGA-AA-A00[illegible]
LATS1_ENST00000253339/ENST00000543571	ENST00000253339	TCGA-BS-A0U[illegible]
LATS1_ENST00000253339/ENST00000543571	ENST00000253339	RK133_C01
LATS1_ENST00000253339/ENST00000543571	ENST00000253339	TCGA-D1-A17[illegible]
LATS1_ENST00000253339/ENST00000543571	ENST00000253339	TCGA-A5-A0G[illegible]
LATS1_ENST00000253339/ENST00000543571	ENST00000253339	TCGA-AZ-659[illegible]
LATS1_ENST00000253339/ENST00000543571	ENST00000253339	267T
LATS1_ENST00000253339/ENST00000543571	ENST00000253339	TCGA-CK-591[illegible]
LATS1_ENST00000253339	ENST00000253339	TCGA-24-160[illegible]
LATS1_ENST00000543571	ENST00000543571	TCGA-A6-267[illegible]
LATS1	ENST00000543571	Br27P
LATS1_ENST00000253339/ENST00000543571	ENST00000253339	TCGA-CK-591[illegible]
LATS1_ENST00000253339/ENST00000543571	ENST00000253339	TCGA-CM-616[illegible]
LATS1_ENST00000253339/ENST00000543571	ENST00000253339	TCGA-E2-A14[illegible]
LATS1_ENST00000253339/ENST00000543571	ENST00000253339	TCGA-AX-A0J[illegible]
LATS1_ENST00000253339/ENST00000543571	ENST00000253339	T155
LATS1_ENST00000543571	ENST00000543571	TCGA-AG-A00[illegible]
LATS1_ENST00000253339/ENST00000543571	ENST00000253339	TCGA-AX-A05[illegible]
LATS1_ENST00000253339/ENST00000543571	ENST00000253339	TCGA-AA-A00[illegible]
LATS1_ENST00000253339	ENST00000253339	TCGA-24-141[illegible]
LATS1_ENST00000253339/ENST00000543571	ENST00000253339	TCGA-69-797[illegible]
LATS1_ENST00000253339/ENST00000543571	ENST00000253339	TCGA-50-659[illegible]
LATS1_ENST00000253339/ENST00000543571	ENST00000253339	YUDAB
LATS1_ENST00000253339/ENST00000543571	ENST00000253339	RK050_C01
LATS1_ENST00000253339/ENST00000543571	ENST00000253339	TCGA-55-790[illegible]
LATS1_ENST00000253339/ENST00000543571	ENST00000253339	TCGA-AA-368[illegible]
LATS1_ENST00000253339/ENST00000543571	ENST00000253339	TCGA-91-682[illegible]
LATS1_ENST00000543571	ENST00000543571	TCGA-AA-351[illegible]

LATS1_ENST00000253339/ENST00000543571	ENST00000253339	TCGA-AD-688
LATS1_ENST00000253339/ENST00000543571	ENST00000253339	TCGA-35-412
LATS1_ENST00000253339/ENST00000543571	ENST00000253339	LUAD-CHTN-3
LATS1_ENST00000253339/ENST00000543571	ENST00000253339	TCGA-A5-A0G
LATS1_ENST00000253339/ENST00000543571	ENST00000253339	HCT-15
LATS1_ENST00000253339/ENST00000543571	ENST00000253339	TCGA-22-460
LATS1_ENST00000253339/ENST00000543571	ENST00000253339	TCGA-CF-A3M
LATS1_ENST00000253339/ENST00000543571	ENST00000253339	TCGA-BS-A0U
LATS1_ENST00000253339/ENST00000543571	ENST00000253339	TCGA-F4-657
LATS1_ENST00000253339/ENST00000543571	ENST00000253339	TCGA-29-169
LATS1_ENST00000253339/ENST00000543571	ENST00000253339	TCGA-D1-A16
LATS1_ENST00000253339/ENST00000543571	ENST00000253339	BN26T
LATS1_ENST00000253339/ENST00000543571	ENST00000253339	TCGA-D8-A14
LATS1_ENST00000253339/ENST00000543571	ENST00000253339	LPJ108
LATS1_ENST00000253339	ENST00000253339	103854
LATS1_ENST00000253339/ENST00000543571	ENST00000253339	CHC1053T
LATS1_ENST00000253339/ENST00000543571	ENST00000253339	ccRCC-41
LATS1_ENST00000253339/ENST00000543571	ENST00000253339	TCGA-AA-A02
LATS1_ENST00000253339/ENST00000543571	ENST00000253339	TCGA-66-278
LATS1_ENST00000253339	ENST00000253339	103450
LATS1_ENST00000253339/ENST00000543571	ENST00000253339	LOXIMVI
LATS1_ENST00000253339/ENST00000543571	ENST00000253339	TCGA-CL-591
LATS1_ENST00000253339/ENST00000543571	ENST00000253339	HCC121T
LATS1_ENST00000253339	ENST00000253339	TCGA-04-133
LATS1_ENST00000253339	ENST00000253339	TCGA-A8-A07
LATS1_ENST00000253339/ENST00000543571	ENST00000253339	TCGA-13-071
LATS1_ENST00000253339/ENST00000543571	ENST00000253339	MCF7
LATS1_ENST00000253339/ENST00000543571	ENST00000253339	TCGA-AA-A00
LATS1_ENST00000543571	ENST00000543571	PD3945a
LATS1_ENST00000253339/ENST00000543571	ENST00000253339	pfg019T
LATS1	ENST00000543571	PD1414a
LATS1	ENST00000543571	PD1364a

SampleID	AAMutation	CDSMutation	PrimaryTissue	TissueSubtype	TissueSubtype	Histology
1780932	p.V25F	c.73G>T	Lung	NS	NS	Carcinoma
1779866	p.R28Q	c.83G>A	Kidney	NS	NS	Carcinoma
1900028	p.E36D	c.108A>T	Breast	NS	NS	Carcinoma
1783377	p.S45Y	c.134C>A	Endometrium	NS	NS	Carcinoma
1919210	p.T59A	c.175A>G	Liver	NS	NS	Carcinoma
1919258	p.T59A	c.175A>G	Liver	NS	NS	Carcinoma
1650967	p.E60G	c.179A>G	Largeintestine	Colon	Ascending	Carcinoma
1558613	p.L78fs*54	c.233delT	Ovary	NS	NS	Carcinoma
1783388	p.R82*	c.244C>T	Endometrium	NS	NS	Carcinoma
1783478	p.R82*	c.244C>T	Endometrium	NS	NS	Carcinoma
2013674	p.R82*	c.244C>T	Skin	NS	NS	Malignantmel
1890935	p.R82Q	c.245G>A	Oesophagus	Lowerthird	NS	Carcinoma
1783388	p.E100*	c.298G>T	Endometrium	NS	NS	Carcinoma
1765262	p.P158S	c.472C>T	Lung	NS	NS	Carcinoma
1898132	p.W178*	c.534G>A	Urinarytract	Bladder	NS	Carcinoma
2062384	p.P193fs*11	c.575_576ins(	NS	NS	NS	Malignantmel
1759179	p.Y200S	c.599A>C	Lung	NS	NS	Carcinoma
1783376	p.R233S	c.699A>C	Endometrium	NS	NS	Carcinoma
1759193	p.V234L	c.700G>C	Lung	NS	NS	Carcinoma
1651148	p.R252G	c.754A>G	Largeintestine	Caecum	NS	Carcinoma
1651142	p.R252G	c.754A>G	Largeintestine	Colon	Ascending	Carcinoma
1423755	p.R252I	c.755G>T	Lung	NS	NS	Carcinoma
1651337	p.T255A	c.763A>G	Largeintestine	Caecum	NS	Carcinoma
1651254	p.T255A	c.763A>G	Largeintestine	Colon	Sigmoid	Carcinoma
1423698	p.T255N	c.764C>A	Lung	NS	NS	Carcinoma
1675361	p.P263S	c.787C>T	Skin	Headneck	NS	Malignantmel
1765245	p.P266fs*19	c.797delC	Lung	NS	NS	Carcinoma
1651595	p.Y277C	c.830A>G	Largeintestine	Rectum	NS	Carcinoma
1779245	p.I288M	c.864C>G	Urinarytract	Bladder	NS	Carcinoma
1651090	p.P301H	c.902C>A	Largeintestine	Colon	Sigmoid	Carcinoma
2013574	p.S308F	c.923C>T	Skin	NS	NS	Malignantmel
1659977	p.S321T	c.962G>C	Breast	NS	NS	Carcinoma
1650923	p.S336G	c.1006A>G	Largeintestine	Caecum	NS	Carcinoma
1779245	p.S387F	c.1160C>T	Urinarytract	Bladder	NS	Carcinoma
1520721	p.G393A	c.1178G>C	Lung	NS	NS	Carcinoma
1520784	p.S394Y	c.1181C>A	Ovary	NS	NS	Other
1914093	p.H417D	c.1249C>G	Lung	Rightupperlol	NS	Carcinoma
1780232	p.M419I	c.1257G>A	Lung	NS	NS	Carcinoma
1914016	p.N463S	c.1388A>G	Lung	Middlelobe	NS	Carcinoma
1780070	p.N471S	c.1412A>G	Lung	NS	NS	Carcinoma

1651333	p.R502C	c.1504C>T	Largeintestine	Caecum	NS	Carcinoma
1651193	p.R502C	c.1504C>T	Largeintestine	Colon	Ascending	Carcinoma
1423693	p.R502C	c.1504C>T	Lung	NS	NS	Carcinoma
1474887	p.P506R	c.1517C>G	Ovary	NS	NS	Carcinoma
1913986	p.W519C	c.1557G>C	Lung	Rightupperlol	NS	Carcinoma
1998442	p.P533fs*8	c.1593delT	Largeintestine	Colon	NS	Carcinoma
1779769	p.G535E	c.1604G>A	Cervix	NS	NS	Carcinoma
1779924	p.G554E	c.1661G>A	Kidney	NS	NS	Carcinoma
1783368	p.P579S	c.1735C>T	Endometrium	NS	NS	Carcinoma
1980746	p.K595N	c.1785G>T	Kidney	NS	NS	Carcinoma
1783352	p.K607N	c.1821G>T	Endometrium	NS	NS	Carcinoma
1779784	p.I615T	c.1844T>C	Kidney	NS	NS	Carcinoma
1863760	p.N620K	c.1860C>A	Lung	NS	NS	Carcinoma
1651102	p.F641L	c.1923C>A	Largeintestine	Caecum	NS	Carcinoma
1783473	p.F641L	c.1923C>A	Endometrium	NS	NS	Carcinoma
1918882	p.E644G	c.1931A>G	Liver	NS	NS	Carcinoma
1783520	p.V650A	c.1949T>C	Endometrium	NS	NS	Carcinoma
1783305	p.R657C	c.1969C>T	Endometrium	NS	NS	Carcinoma
1651172	p.K662fs*3	c.1986delA	Largeintestine	Caecum	NS	Carcinoma
2067177	p.Q663L	c.1988A>T	Liver	NS	NS	Carcinoma
1651200	p.E689fs*18	c.2064delA	Largeintestine	Caecum	NS	Carcinoma
1475042	p.R694C	c.2080C>T	Ovary	NS	NS	Carcinoma
1650923	p.M704V	c.2110A>G	Largeintestine	Caecum	NS	Carcinoma
1312984	p.V719I	c.2155G>A	Centralnervou	Brain	NS	Glioma
1651200	p.V719A	c.2156T>C	Largeintestine	Caecum	NS	Carcinoma
1651230	p.V719A	c.2156T>C	Largeintestine	Colon	Ascending	Carcinoma
1900113	p.R737*	c.2209C>T	Breast	NS	NS	Carcinoma
1783376	p.R737*	c.2209C>T	Endometrium	NS	NS	Carcinoma
1861137	p.R737*	c.2209C>T	Endometrium	NS	NS	Carcinoma
1651648	p.R737*	c.2209C>T	Largeintestine	Rectum	NS	Carcinoma
1783365	p.R744*	c.2230C>T	Endometrium	NS	NS	Carcinoma
1651102	p.R744Q	c.2231G>A	Largeintestine	Caecum	NS	Carcinoma
1475026	p.A748T	c.2242G>A	Ovary	NS	NS	Carcinoma
1914043	p.A758S	c.2271_2272G	Lung	Rightupperlol	NS	Carcinoma
1780168	p.N762S	c.2285A>G	Lung	NS	NS	Carcinoma
2013596	p.M782I	c.2346G>A	Skin	NS	NS	Malignantmel
1918840	p.P786H	c.2357C>A	Liver	NS	NS	Carcinoma
1914028	p.G787V	c.2360G>T	Lung	Rightupperlol	NS	Carcinoma
1651021	p.M790T	c.2369T>C	Largeintestine	Caecum	NS	Carcinoma
1780235	p.S803T	c.2408G>C	Lung	NS	NS	Carcinoma
1650977	p.A805V	c.2414C>T	Largeintestine	Colon	Ascending	Carcinoma

1651142	p.R806*	c.2416C>T	Largeintestine	Colon	Ascending	Carcinoma
1780086	p.H820R	c.2459A>G	Lung	NS	NS	Carcinoma
1765183	p.R827T	c.2480G>C	Lung	NS	NS	Carcinoma
1783305	p.L844M	c.2530T>A	Endometrium	NS	NS	Carcinoma
1998443	p.T851I	c.2552C>T	Largeintestine	Colon	NS	Carcinoma
1780891	p.R854K	c.2561G>A	Lung	NS	NS	Carcinoma
1898088	p.Q863E	c.2587C>G	Urinarytract	Bladder	NS	Carcinoma
1783478	p.D871Y	c.2611G>T	Endometrium	NS	NS	Carcinoma
1651303	p.P882H	c.2645C>A	Largeintestine	Colon	Transverse	Carcinoma
1731238	p.L894I	c.2680T>A	Ovary	NS	NS	Carcinoma
1783500	p.A899fs*5	c.2695_2704d	Endometrium	NS	NS	Carcinoma
1919146	p.T913I	c.2738C>T	Liver	NS	NS	Carcinoma
1900046	p.E920fs*9	c.2759_2769d	Breast	NS	NS	Carcinoma
1901068	p.F950L	c.2850C>A	Haematopoie	NS	NS	Lymphoidneo
1520441	p.Q959H	c.2877A>C	Breast	NS	NS	Carcinoma
1713734	p.?	c.2883+1G>T	Liver	NS	NS	Carcinoma
1980777	p.P973T	c.2917C>A	Kidney	NS	NS	Carcinoma
1651130	p.R990Q	c.2969G>A	Largeintestine	Colon	Sigmoid	Carcinoma
1782843	p.D994N	c.2980G>A	Lung	NS	NS	Carcinoma
1520559	p.R995C	c.2983C>T	Breast	NS	NS	Carcinoma
1998452	p.R995L	c.2984G>T	Haematopoie	NS	NS	Lymphoidneo
1651598	p.R995H	c.2984G>A	Largeintestine	Rectum	NS	Carcinoma
1919180	p.F1010L	c.3030T>A	Liver	NS	NS	Carcinoma
1474885	p.R1020T	c.3059G>C	Ovary	NS	NS	Carcinoma
1899686	p.P1028A	c.3082C>G	Breast	NS	NS	Carcinoma
1474912	p.P1028T	c.3082C>A	Ovary	NS	NS	Carcinoma
1998454	p.D1043N	c.3127G>A	Breast	NS	NS	Carcinoma
1651102	p.V1057A	c.3170T>C	Largeintestine	Caecum	NS	Carcinoma
1284069	p.Q1116E	c.3346C>G	Breast	NS	NS	Carcinoma
1943747	p.R1125C	c.3373C>T	Stomach	NS	NS	Adenocarcino
895764	p.M669I	c.2007G>A	Lung	NS	NS	Carcinoma
753392	p.R806P	c.2417G>C	Lung	NS	NS	Carcinoma

HistologySubt	HistologySubt	PubmedId	CGPStudy	SomaticStatus	SampleSource	Zygosity
Squamouscell	NS	-	COSU418	ConfirmedSor	TumourSampl	Unknown
Clearcellrenal	NS	-	COSU416	ConfirmedSor	TumourSampl	Unknown
NS	NS	-	COSU414	Variantofunkr	TumourSampl	Heterozygous
Endometrioid	NS	-	COSU419	Variantofunkr	TumourSampl	Heterozygous
NS	NS	-	COSU323	Variantofunkr	TumourSampl	Heterozygous
NS	NS	-	COSU323	Variantofunkr	TumourSampl	Heterozygous
Adenocarcino	NS	-	COSU376	Variantofunkr	Unknown	Heterozygous
Serouscarcinc	NS	21720365	COSU331	ConfirmedSor	Unknown	Heterozygous
Endometrioid	NS	-	COSU419	PreviouslyRep	TumourSampl	Heterozygous
Endometrioid	NS	-	COSU419	PreviouslyRep	TumourSampl	Heterozygous
NS	NS	22842228	COSU511	ConfirmedSor	Unknown	Heterozygous
Adenocarcino	NS	23525077	COSU464	Variantofunkr	TumourSampl	Unknown
Endometrioid	NS	-	COSU419	Variantofunkr	TumourSampl	Heterozygous
Adenocarcino	NS	22980975	COSU431	Variantofunkr	TumourSampl	Unknown
NS	NS	-	COSU413	Variantofunkr	TumourSampl	Unknown
NS	NS	24265154	COSU526	ConfirmedSor	Unknown	Homozygous
Smallcellcarci	NS	22941188	COSU423	ConfirmedSor	TumourSampl	Unknown
Endometrioid	NS	-	COSU419	Variantofunkr	TumourSampl	Heterozygous
Smallcellcarci	NS	22941188	COSU423	Variantofunkr	TumourSampl	Unknown
Adenocarcino	NS	-	COSU376	Variantofunkr	Unknown	Heterozygous
Adenocarcino	NS	-	COSU376	Variantofunkr	Unknown	Heterozygous
Adenocarcino	NS	18948947	COSU341	ConfirmedSor	Unknown	Heterozygous
Adenocarcino	NS	-	COSU376	Variantofunkr	Unknown	Heterozygous
Adenocarcino	NS	-	COSU376	Variantofunkr	Unknown	Heterozygous
Adenocarcino	NS	18948947	COSU341	ConfirmedSor	Unknown	Heterozygous
Superficialspr	NS	22197931	COSU389	Variantofunkr	Cultured	Heterozygous
Adenocarcino	NS	22980975	COSU431	Variantofunkr	TumourSampl	Unknown
Adenocarcino	NS	-	COSU375	Variantofunkr	TumourSampl	Heterozygous
NS	NS	-	COSU413	ConfirmedSor	TumourSampl	Unknown
Adenocarcino	NS	-	COSU376	ConfirmedSor	Unknown	Unknown
NS	NS	22842228	COSU511	ConfirmedSor	Unknown	Heterozygous
Basal(triple-ne	NS	22495314	COSU384	ConfirmedSor	TumourSampl	Unknown
Adenocarcino	Mucinous	-	COSU376	ConfirmedSor	Unknown	Unknown
NS	NS	-	COSU413	ConfirmedSor	TumourSampl	Unknown
Squamouscell	NS	20668451	COSU338	ConfirmedSor	TumourSampl	Unknown
Neoplasm	NS	20668451	COSU338	ConfirmedSor	TumourSampl	Unknown
Adenocarcino	NS	-	COSU417	Variantofunkr	TumourSampl	Unknown
Adenocarcino	NS	-	COSU417	Variantofunkr	TumourSampl	Unknown
Adenocarcino	NS	-	COSU417	Variantofunkr	TumourSampl	Unknown
Adenocarcino	NS	-	COSU417	Variantofunkr	TumourSampl	Unknown

Adenocarcino	NS	-	COSU376	Variantofunkr	Unknown	Heterozygous
Adenocarcino	NS	-	COSU376	Variantofunkr	Unknown	Heterozygous
Adenocarcino	NS	18948947	COSU341	ConfirmedSor	Unknown	Heterozygous
Serouscarcinc	NS	21720365	COSU331	ConfirmedSor	TumourSampl	Heterozygous
Adenocarcino	NS	-	COSU417	Variantofunkr	TumourSampl	Unknown
NS	NS	23856246	COSU504	ConfirmedSor	Cultured	Heterozygous
Squamouscell	NS	-	COSU415	ConfirmedSor	TumourSampl	Unknown
Clearcellrenal	NS	-	COSU416	ConfirmedSor	TumourSampl	Unknown
Endometrioid	NS	-	COSU419	Variantofunkr	TumourSampl	Heterozygous
Clearcellrenal	NS	23797736	COSU494	ConfirmedSor	Unknown	Unknown
Endometrioid	NS	-	COSU419	Variantofunkr	TumourSampl	Heterozygous
Clearcellrenal	NS	-	COSU416	ConfirmedSor	TumourSampl	Unknown
Adenocarcino	NS	22975805	COSU453	ConfirmedSor	TumourSampl	Unknown
Adenocarcino	Mucinous	22810696	COSU376	ConfirmedSor	Unknown	Unknown
Endometrioid	NS	-	COSU419	PreviouslyRep	TumourSampl	Heterozygous
NS	NS	-	COSU322	Variantofunkr	TumourSampl	Heterozygous
Endometrioid	NS	-	COSU419	ConfirmedSor	TumourSampl	Unknown
Endometrioid	NS	-	COSU419	ConfirmedSor	TumourSampl	Unknown
Adenocarcino	NS	-	COSU376	Variantofunkr	Unknown	Heterozygous
Hepatocellula	NS	23788652	COSU527	ConfirmedSor	TumourSampl	Unknown
Adenocarcino	NS	-	COSU376	Variantofunkr	Unknown	Heterozygous
Serouscarcinc	NS	21720365	COSU331	ConfirmedSor	TumourSampl	Heterozygous
Adenocarcino	Mucinous	-	COSU376	ConfirmedSor	Unknown	Unknown
AstrocytomaG	Glioblastomar	18772396	-	ConfirmedSor	TumourSampl	Heterozygous
Adenocarcino	NS	-	COSU376	Variantofunkr	Unknown	Heterozygous
Adenocarcino	NS	-	COSU376	Variantofunkr	Unknown	Heterozygous
NS	NS	-	COSU414	Variantofunkr	TumourSampl	Heterozygous
Endometrioid	NS	-	COSU419	Variantofunkr	TumourSampl	Heterozygous
Serouscarcinc	NS	23104009	COSU449	Variantofunkr	TumourSampl	Unknown
Adenocarcino	NS	-	COSU375	ConfirmedSor	TumourSampl	Unknown
Endometrioid	NS	-	COSU419	Variantofunkr	TumourSampl	Heterozygous
Adenocarcino	Mucinous	22810696	COSU376	ConfirmedSor	Unknown	Unknown
Serouscarcinc	NS	21720365	COSU331	ConfirmedSor	TumourSampl	Heterozygous
Adenocarcino	NS	-	COSU417	Variantofunkr	TumourSampl	Unknown
Adenocarcino	NS	-	COSU417	Variantofunkr	TumourSampl	Unknown
NS	NS	22842228	COSU511	ConfirmedSor	Unknown	Heterozygous
NS	NS	-	COSU322	Variantofunkr	TumourSampl	Heterozygous
Adenocarcino	NS	-	COSU417	Variantofunkr	TumourSampl	Unknown
Adenocarcino	NS	22810696	COSU376	ConfirmedSor	Unknown	Unknown
Adenocarcino	NS	-	COSU417	Variantofunkr	TumourSampl	Unknown
Adenocarcino	Mucinous	-	COSU376	ConfirmedSor	Unknown	Unknown

Adenocarcino	NS	-		COSU376	Variantofunkr	Unknown	Heterozygous
Adenocarcino	NS	-		COSU417	Variantofunkr	TumourSampl	Unknown
Adenocarcino	NS		22980975	COSU431	Variantofunkr	TumourSampl	Unknown
Endometrioid	NS	-		COSU419	ConfirmedSor	TumourSampl	Unknown
NS	NS		23856246	COSU504	ConfirmedSor	Cultured	Heterozygous
Squamouscell	NS	-		COSU418	ConfirmedSor	TumourSampl	Unknown
NS	NS	-		COSU413	Variantofunkr	TumourSampl	Unknown
Endometrioid	NS	-		COSU419	Variantofunkr	TumourSampl	Heterozygous
Adenocarcino	NS	-		COSU376	Variantofunkr	Unknown	Heterozygous
Serouscarcino	NS	-		COSU331	Variantofunkr	TumourSampl	Heterozygous
Endometrioid	NS	-		COSU419	Variantofunkr	TumourSampl	Heterozygous
NS	NS	-		COSU323	Variantofunkr	TumourSampl	Heterozygous
NS	NS	-		COSU414	Variantofunkr	TumourSampl	Heterozygous
Follicularlymp	NS		23297126	COSU467	ConfirmedSor	TumourSampl	Unknown
HER-positivec	NS		20668451	COSU338	ConfirmedSor	TumourSampl	Unknown
Hepatocellula	NS		22561517	COSU396	Variantofunkr	TumourSampl	Unknown
Clearcellrenal	NS		23797736	COSU494	ConfirmedSor	Unknown	Unknown
Adenocarcino	NS		22810696	COSU376	ConfirmedSor	Unknown	Unknown
Squamouscell	NS	-		COSU418	ConfirmedSor	TumourSampl	Unknown
Basal(triple-ne	NS		20668451	COSU338	ConfirmedSor	TumourSampl	Unknown
Hairycellleuka	NS		23856246	COSU504	ConfirmedSor	Cultured	Heterozygous
Adenocarcino	NS	-		COSU375	Variantofunkr	TumourSampl	Heterozygous
NS	NS	-		COSU323	Variantofunkr	TumourSampl	Heterozygous
Serouscarcino	NS		21720365	COSU331	ConfirmedSor	TumourSampl	Heterozygous
NS	NS	-		COSU414	Variantofunkr	TumourSampl	Heterozygous
Serouscarcino	NS		21720365	COSU331	ConfirmedSor	TumourSampl	Heterozygous
NS	NS		23856246	COSU504	ConfirmedSor	Cultured	Heterozygous
Adenocarcino	Mucinous		22810696	COSU376	ConfirmedSor	Unknown	Unknown
NS	NS		22608084	COSU385	ConfirmedSor	TumourSampl	Unknown
NS	NS		22037554	COSU479	Variantofunkr	TumourSampl	Unknown
Adenocarcino	NS		16140923	COSU22;COSU	ConfirmedSor	TumourSampl	Unknown
Largecellcarci	NS		16140923	COSU22;COSU	ConfirmedSor	TumourSampl	Unknown

GenomicCo-ordinatesGRCh3	SIFT	PROVEAN	PolyPhen-2	MutationAss	SNV in dbSN
6:150023190..150023190	Damaging	Neutral	neutral	benign	
6:150023180..150023180	Tolerated	Neutral	neutral	benign	
6:150023155..150023155	Damaging	Neutral	medium	benign	
6:150023129..150023129	Damaging	Neutral	probablydama	medium	
6:150023088..150023088	Tolerated	Neutral	benign	neutral	
6:150023088..150023088	Tolerated	Neutral	benign	neutral	
6:150023084..150023084	Damaging	Neutral	low	olydamaging/benign	
6:150023030..150023030					
6:150023019..150023019					
6:150023019..150023019					
6:150023019..150023019					
6:150023018..150023018	Damaging	Neutral	medium	blydamaging/benign	
6:150022965..150022965					
6:150016234..150016234	Damaging	Neutral	low	benign	
6:150005691..150005691					
6:150005649..150005650					
6:150005626..150005626	Damaging	Neutral	low	ossiblydamaging	
6:150005526..150005526	Damaging	Neutral	probablydama	low	
6:150005525..150005525	Tolerated	Neutral	low	obablydamaging	
6:150005471..150005471	Damaging	Neutral	low	benign	
6:150005471..150005471	Damaging	Neutral	low	benign	
6:150005470..150005470	Damaging	Neutral	low	blydamaging/benign	
6:150005462..150005462	Tolerated	Neutral	low	benign	
6:150005462..150005462	Tolerated	Neutral	low	benign	
6:150005461..150005461	Tolerated	Neutral	low	benign	
6:150005438..150005438	Tolerated	Neutral	low	obablydamaging	
6:150005428..150005428					
6:150005395..150005395	Damaging	Deleterious	low	obablydamaging	
6:150005361..150005361	Tolerated	Neutral	neutral	benign	
6:150005323..150005323	Damaging	Neutral	low	oly/possiblydamaging	
6:150005302..150005302	Damaging	Neutral	possiblydamag	neutral	
6:150005263..150005263	Damaging	Neutral	low	benign	
6:150005219..150005219	Tolerated	Neutral	neutral	benign	
6:150005065..150005065	Damaging	Neutral	neutral	benign	
6:150005047..150005047	Tolerated	Neutral	low	benign	
6:150005044..150005044	Damaging	Neutral	low	blydamaging/benign	
6:150004976..150004976	Tolerated	Neutral	low	obablydamaging	
6:150004968..150004968	Damaging	Neutral	low	benign	
6:150004837..150004837	Tolerated	Neutral	low	benign	
6:150004813..150004813	Tolerated	Neutral	benign	neutral	

6:150004721..150004721	Damaging	Deleterious	medium	obablydamaging	
6:150004721..150004721	Damaging	Deleterious	medium	obablydamaging	
6:150004721..150004721	Damaging	Deleterious	medium	obablydamaging	
6:150004708..150004708	Damaging	Deleterious	probablydamag	medium	
6:150004668..150004668	Damaging	Deleterious	medium	obablydamaging	
6:150004632..150004632					
6:150004621..150004621	Tolerated	Neutral	low	benign	
6:150004564..150004564	Damaging	Neutral	low	obablydamaging	
6:150004490..150004490	Tolerated	Neutral	benign	neutral	
6:150004440..150004440	Damaging	Neutral	benign	low	
6:150004404..150004404	Damaging	Neutral	benign	medium	
6:150004381..150004381	Damaging	Deleterious	neutral	benign	
6:150004365..150004365	Tolerated	Neutral	low	benign	
6:150004302..150004302	Tolerated	Deleterious	medium	benign	F641L3C>A
6:150004302..150004302	Tolerated	Deleterious	medium	benign	F641L3C>A
6:150004294..150004294	Damaging	Deleterious	probablydamag	medium	
6:150004276..150004276	Tolerated	Deleterious	medium	benign	
6:150004256..150004256	Damaging	Deleterious	medium	obablydamaging	
6:150004239..150004239					
6:150004237..150004237	Damaging	Deleterious	benign	medium	
6:150001540..150001540					
6:150001524..150001524	Damaging	Deleterious	medium	obablydamaging	
6:150001494..150001494	Damaging	Deleterious	neutral	blydamaging/b	M704V,A>G
6:150001449..150001449	Damaging	Neutral	medium	obablydamaging	
6:150001448..150001448	Damaging	Deleterious	medium	obablydamaging	
6:150001448..150001448	Damaging	Deleterious	medium	obablydamaging	
6:150001395..150001395					
6:150001395..150001395					
6:150001395..150001395					
6:150001395..150001395					
6:150001374..150001374					
6:150001373..150001373	Damaging	Deleterious	neutral	obablydamagi	R744Q,G>A
6:150001362..150001362	Damaging	Deleterious	neutral	ly/possiblydamaging	
6:150001332..150001333	Damaging	Deleterious	neutral	ly/possiblydamaging	
6:150001319..150001319	Tolerated	Deleterious	neutral	n/possiblydamaging	
6:150001258..150001258	Damaging	Deleterious	probablydamag	low	
6:150001247..150001247	Damaging	Deleterious	probablydamag	low	
6:150001244..15000124	Damaging	Deleterious	medium	obablydamaging	
6:150001235..150001235	Damaging	Deleterious	low	obablydamaging	
6:150001196..150001196	Tolerated	Neutral	neutral	benign	
6:150001190..150001190	Damaging	Deleterious	neutral	obablydamaging	

6:150001188..150001188					
6:150001145..150001145	Damaging	Deleterious	medium	obablydamaging	
6:150001124..150001124	Damaging	Deleterious	medium	obablydamaging	
6:150001074..150001074	Damaging	Neutral	medium	obablydamaging	
6:150001052..150001052	Damaging	Deleterious	probablydama	low	
6:150001043..150001043	Tolerated	Deleterious	neutral	gn/possiblydamaging	
6:150001017..150001017	Tolerated	Deleterious	neutral	benign	
6:149997856..149997856	Damaging	Deleterious	probably/poss	low	
6:149997822..149997822	Tolerated	Neutral	low	bly/possiblydamaging	
6:149997787..149997787	Damaging	Neutral	low	ossiblydamaging	
6:149997763..149997772					
6:149997729..149997729	Damaging	Deleterious	probablydamag	medium	
6:149997698..149997708					
6:149997429..149997429	Damaging	Deleterious	medium	obablydamaging	
6:149997402..149997402	Damaging	Deleterious	low	obablydamaging	
6:149997395..149997395					
6:149983341..149983341	Tolerated	Neutral	benign	low	
6:149983289..149983289	Damaging	Neutral	neutral	blydamaging/benign	
6:149983278..149983278	Tolerated	Neutral	neutral	benign	
6:149983275..149983275	Damaging	Deleterious	medium	obablydamaging	
6:149983274..149983274	Damaging	Deleterious	probablydama	medium	
6:149983274..149983274	Damaging	Deleterious	medium	obablydamaging	
6:149983228..149983228	Damaging	Deleterious	probablydamag	low	
6:149983199..149983199	Damaging	Deleterious	medium	obablydamaging	
6:149983176..149983176	Damaging	Deleterious	medium	obablydamaging	
6:149983176..149983176	Damaging	Deleterious	high	obablydamaging	
6:149983131..149983131	Damaging	Deleterious	probablydamag	medium	
6:149983088..149983088	Damaging	Neutral	neutral	benign	
6:149982912..149982912	Damaging	Neutral	neutral	benign	
6:149982885..149982885	Tolerated	Neutral	benign	neutral	R1125C,C>T
-	Tolerated	Neutral	neutral	benign	
-	Damaging	Deleterious	low	obablydamaging	

MAF(Minor Allel Frequency) in dbSNP

0.0005
