# Supplementary material for: Mutation analysis of large tumor suppressor genes LATS1 and LATS2 supports a tumor suppressor role in human cancer
Source: Protein Cell. 2014 Dec 9;6(1):6–11. doi: 10.1007/s13238-014-0122-4 (PMC4286129; doi:10.1007/s13238-014-0122-4)
Supplement: Supplementary file 3 — Supplementary material 3 (PDF 358 kb) [file 13238_2014_122_MOESM3_ESM.pdf]

| Cancer Study | Type | AA Change |
|---|---|---|
| G361_SKIN | CCLE (Broad) | *1089Y |
| TCGA-AN-A0XN | Breast (TCGA pub) | A472_480APAPAPAPA>A |
| P05-1657 | Prostate (Broad/Cornell 2( | P479_479P>PAP |
| TCGA-IB-7897 | Panceas (TCGA) | P479_479P>PAP |
| TCGA-J7-8537 | pRCC (TCGA) | P479_479P>PAP |
| TCGA-EJ-7784 | Prostate (TCGA) | P479_479P>PAP |
| TCGA-AX-A0J1 | Uterine (TCGA pub) | A110T |
| TCGA-AX-A0J1 | Uterine (TCGA) | A110T |
| ESO-114 | Esophagus (Broad) | A120G |
| TCGA-BR-6452 | Stomach (TCGA) | A309V |
| TCGA-A1-A0SJ | Breast (TCGA pub) | A324V |
| TCGA-D1-A103 | Uterine (TCGA) | A392fs |
| TCGA-D1-A103 | Uterine (TCGA pub) | A392fs |
| TCGA-D1-A103 | Uterine (TCGA pub) | A428V |
| TCGA-D1-A103 | Uterine (TCGA) | A428V |
| TCGA-B5-A11J | Uterine (TCGA pub) | A497T |
| TCGA-B5-A11J | Uterine (TCGA) | A497T |
| TCGA-BR-6452 | Stomach (TCGA) | A546V |
| TCGA-AG-A002 | Colorectal (TCGA pub) | A561T |
| TCGA-AG-A002 | Colorectal (TCGA) | A561T |
| SUDHL10_HAEMATOP... | CCLE (Broad) | A678S |
| TCGA-B5-A11E | Uterine (TCGA pub) | A773T |
| TCGA-B5-A11E | Uterine (TCGA) | A773T |
| TCGA-BR-4184 | Stomach (TCGA) | A861V |
| BR-V-032 | Breast (Broad) | A861V |
| TCGA-AA-3525 | Colorectal (TCGA) | A881V |
| TCGA-AA-3525 | Colorectal (TCGA pub) | A881V |
| SNU1040_LARGE_IN... | CCLE (Broad) | A944V |
| TCGA-50-5044 | Lung adeno (TCGA pub) | C1083Y |
| TCGA-50-5044 | Lung adeno (TCGA) | C1083Y |
| H070599 | Liver (LGGM) | D1048N |
| NCIH1703_LUNG | CCLE (Broad) | D1078Y |
| NCIH1930_LUNG | CCLE (Broad) | D564Y |
| RKO_LARGE_INTEST... | CCLE (Broad) | D569G |
| TCGA-B5-A0JY | Uterine (TCGA) | D56Y |
| TCGA-B5-A0JY | Uterine (TCGA pub) | D56Y |
| TCGA-AN-A046 | Breast (TCGA) | D800Y |
| PD4120a | Breast (Sanger) | D852N |
| 16929 | Lung adeno (TSP) | E1016K |
| TCGA-BS-A0UV | Uterine (TCGA pub) | E1039K |

| | | |
|---|---|---|
| TCGA-BS-A0UV | Uterine (TCGA) | E1039K |
| SA214 | Breast (BCCRC) | E1067A |
| TCGA-CN-4723 | Head & neck (TCGA pub) | E130K |
| TCGA-CN-4723 | Head & neck (TCGA) | E130K |
| TCGA-NA-A4R1 | Uterine CS (TCGA) | E591fs |
| TCGA-AN-A046 | Breast (TCGA) | E652* |
| TCGA-B0-5711 | ccRCC (TCGA pub) | E722* |
| TCGA-B0-5711 | ccRCC (TCGA) | E722* |
| TCGA-BP-4326 | ccRCC (TCGA) | E726K |
| TCGA-BP-4326 | ccRCC (TCGA pub) | E726K |
| HCT15_LARGE_INTE... | CCLE (Broad) | E765D |
| HCT_15 | NCI-60 | E765D |
| HRT18_LARGE_INTE... | CCLE (Broad) | E765D |
| TCGA-BR-8680 | Stomach (TCGA) | F810S |
| JHUEM7_ENDOMETRIUM | CCLE (Broad) | F972L |
| TCGA-05-4424 | Lung adeno (TCGA) | G218V |
| TCGA-05-4424 | Lung adeno (TCGA pub) | G218V |
| IGROV1_OVARY | CCLE (Broad) | G293D |
| TCGA-AO-A12B | Breast (TCGA pub) | G363S |
| MOTN1_HAEMATOPOI... | CCLE (Broad) | G36W |
| NCIH2126_LUNG | CCLE (Broad) | G40E |
| TCGA-DU-7300 | Glioma (TCGA) | G498V |
| MM-0521 | MM (Broad) | G539D |
| P05-3852 | Prostate (Broad/Cornell 20 | G566C |
| TCGA-44-6774 | Lung adeno (TCGA pub) | G803C |
| TCGA-44-6774 | Lung adeno (TCGA) | G803C |
| TCGA-EE-A20H | Melanoma (TCGA) | G851E |
| MEL-JWCI-WGS-4 | Melanoma (Broad) | G92S |
| TCGA-CG-5721 | Stomach (TCGA) | H317Y |
| TCGA-CJ-5684 | ccRCC (TCGA pub) | H691fs |
| TCGA-CJ-5684 | ccRCC (TCGA) | H691fs |
| TCGA-B5-A0JZ | Uterine (TCGA) | H970N |
| TCGA-B5-A0JZ | Uterine (TCGA pub) | H970N |
| MM-0529 | MM (Broad) | I149T |
| TCGA-BG-A0MU | Uterine (TCGA) | I80fs |
| TCGA-BG-A0MU | Uterine (TCGA pub) | I80fs |
| OVISE_OVARY | CCLE (Broad) | I902L |
| GP2D_LARGE_INTES... | CCLE (Broad) | I902N |
| ESO-0013 | Esophagus (Broad) | K665R |
| H050227 | Liver (LGGM) | K702M |
| BEN_LUNG | CCLE (Broad) | L625V |

| | | | |
|---|---|---|---|
| TCGA-B5-A11E | | Uterine (TCGA) | L693M |
| TCGA-B5-A11E | | Uterine (TCGA pub) | L693M |
| HSC3_UPPER_AEROD... | | CCLE (Broad) | L699V |
| LOUNH91_LUNG | | CCLE (Broad) | L77fs |
| TCGA-B5-A11E | | Uterine (TCGA) | L841F |
| TCGA-B5-A11E | | Uterine (TCGA pub) | L841F |
| TCGA-AA-3555 | | Colorectal (TCGA) | L903I |
| TCGA-AA-3555 | | Colorectal (TCGA pub) | L903I |
| KM12_LARGE_INTES... | | CCLE (Broad) | L914fs |
| TCGA-AP-A059 | | Uterine (TCGA) | L967M |
| TCGA-AP-A059 | | Uterine (TCGA pub) | L967M |
| TCGA-BR-4361 | | Stomach (TCGA) | N725S |
| TCGA-FW-A3R5 | | Melanoma (TCGA) | P166S |
| TCGA-D6-6516 | | Head & neck (TCGA) | P190S |
| TCGA-D6-6516 | | Head & neck (TCGA pub) | P190S |
| | 587284 | Colorectal (Genentech) | P208L |
| TCGA-BR-4368 | | Stomach (TCGA) | P210S |
| MEL-Ma-Mel-114 | | Melanoma (Broad) | P305L |
| LOVO_LARGE_INTES... | | CCLE (Broad) | P516L |
| TCGA-18-3409 | | Lung squ (TCGA) | P516L |
| TCGA-18-3409 | | Lung squ (TCGA pub) | P516L |
| DOHH2_HAEMATOPOI... | | CCLE (Broad) | P551L |
| TCGA-EE-A29L | | Melanoma (TCGA) | P577L |
| TCGA-36-1568 | | Ovarian (TCGA pub) | P72L |
| TCGA-64-5775 | | Lung adeno (TCGA) | P996L |
| TCGA-64-5775 | | Lung adeno (TCGA pub) | P996L |
| LUAD-5V8LT | | Lung adeno (Broad) | Q105P |
| RT11284_URINARY_... | | CCLE (Broad) | Q1079E |
| TCGA-CG-5721 | | Stomach (TCGA) | Q345* |
| TCGA-55-1594 | | Lung adeno (TCGA) | Q643E |
| TCGA-55-1594 | | Lung adeno (TCGA pub) | Q643E |
| DMS153_LUNG | | CCLE (Broad) | Q74R |
| TCGA-IB-7651 | | Panceas (TCGA) | R1043* |
| TCGA-D1-A17Q | | Uterine (TCGA pub) | R1054* |
| TCGA-D1-A17Q | | Uterine (TCGA) | R1054* |
| MUTZ5_HAEMATOPOI... | | CCLE (Broad) | R1054Q |
| SNU175_LARGE_INT... | | CCLE (Broad) | R18* |
| NCIH889_LUNG | | CCLE (Broad) | R271H |
| | 587342 | Colorectal (Genentech) | R391H |
| | 587278 | Colorectal (Genentech) | R415W |

| | | |
|---|---|---|
| HPBALL_HAEMATOPO... | CCLE (Broad) | R525C |
| SNU175_LARGE_INT... | CCLE (Broad) | R525C |
| SNU1_STOMACH | CCLE (Broad) | R525C |
| TCGA-BT-A20J | Bladder (TCGA) | R525C |
| TCGA-BT-A20J | Bladder (TCGA pub) | R525C |
| TCGA-BT-A0YX | Bladder (TCGA pub) | R558H |
| TCGA-B5-A11E | Uterine (TCGA pub) | R558H |
| TCGA-A5-A0VQ | Uterine (TCGA) | R581C |
| TCGA-A5-A0VQ | Uterine (TCGA pub) | R581C |
| TCGA-FG-A60J | Glioma (TCGA) | R593C |
| TCGA-EE-A3JE | Melanoma (TCGA) | R623W |
| TCGA-05-4396 | Lung adeno (TCGA) | R645L |
| KS1_CENTRAL_NERV... | CCLE (Broad) | R759W |
| HCT_15 | NCI-60 | R769W |
| HCT15_LARGE_INTE... | CCLE (Broad) | R769W |
| HRT18_LARGE_INTE... | CCLE (Broad) | R769W |
| TCGA-KN-8427 | chRCC (TCGA) | R790Q |
| CCK81_LARGE_INTE... | CCLE (Broad) | R817G |
| TCGA-FP-7735 | Stomach (TCGA) | R832G |
| H091794 | Liver (LGGM) | R849L |
| H090284 | Liver (LGGM) | R983L |
| TCGA-66-2787 | Lung squ (TCGA pub) | R983L |
| TCGA-AN-A046 | Breast (TCGA) | S179L |
| TCGA-AG-A002 | Colorectal (TCGA) | S33L |
| TCGA-AG-A002 | Colorectal (TCGA pub) | S33L |
| ME033 | Melanoma (Broad) | S366F |
| TCGA-46-3769 | Lung squ (TCGA) | S528L |
| TCGA-46-3769 | Lung squ (TCGA pub) | S528L |
| TCGA-CW-5589 | ccRCC (TCGA pub) | S596R |
| TCGA-CW-5589 | ccRCC (TCGA) | S596R |
| AN3CA_ENDOMETRIUM | CCLE (Broad) | S872L |
| TCGA-AP-A056 | Uterine (TCGA pub) | S91L |
| TCGA-AP-A056 | Uterine (TCGA) | S91L |
| HCT15_LARGE_INTE... | CCLE (Broad) | T1041I |
| HCT_15 | NCI-60 | T1041I |
| TCGA-A2-A0T5 | Breast (TCGA) | T1041P |
| TCGA-AP-A0LM | Uterine (TCGA pub) | T168M |
| TCGA-AP-A0LM | Uterine (TCGA) | T168M |
| TCGA-BR-8372 | Stomach (TCGA) | T168M |
| TCGA-BR-4362 | Stomach (TCGA) | T168M |
| TCGA-FW-A3R5 | Melanoma (TCGA) | T673I |

| | | |
|---|---|---|
| SNU81_LARGE_INTE... | CCLE (Broad) | T876N |
| GP2D_LARGE_INTES... | CCLE (Broad) | V1086A |
| TCGA-44-7670 | Lung adeno (TCGA pub) | V682L |
| TCGA-HT-8113 | Glioma (TCGA) | V729D |
| SW948_LARGE_INTE... | CCLE (Broad) | W842L |
| LUAD-YINHD | Lung adeno (Broad) | Y183C |
| SHP77_LUNG | CCLE (Broad) | Y506F |
| MDAPCA2B_PROSTATE | CCLE (Broad) | Y531H |

| Cosmic | FIS | Cons | 3D | VS | Allele Frequen | #Mut in Sampl |
|---|---|---|---|---|---|---|
| Nonstop | diploid | 7 | U | | 0.65 | 47 |
| IF del | gain | | U | | NA | 111 |
| IF ins | diploid | 1 | U | | NA | 27 |
| IF ins | diploid | 1 | U | | 0.27 | 206 |
| IF ins | hetloss | 1 | U | | 0.31 | 41 |
| IF ins | gain | 1 | U | | 0.5 | 60 |
| Missense | diploid | 1 | U | Neutral | 0.35 | 3848 |
| Missense | diploid | 1 | U | Neutral | 0.35 | 3747 |
| Missense | NA | 1 | U | Neutral | 0.45 | 335 |
| Missense | diploid | | U | Neutral | 0.23 | 6281 |
| Missense | diploid | 1 | U | Neutral | NA | 29 |
| FS del | diploid | 7 | U | | NA | 5781 |
| FS del | diploid | 7 | U | | NA | 5965 |
| Missense | diploid | 1 | U | Neutral | 0.5 | 5965 |
| Missense | diploid | 1 | U | Neutral | 0.5 | 5781 |
| Missense | diploid | 1 | U | Neutral | 0.38 | 318 |
| Missense | diploid | 1 | U | Neutral | 0.38 | 309 |
| Missense | diploid | | U | Neutral | 0.05 | 6281 |
| Missense | diploid | 1 | V | Neutral | NA | 9896 |
| Missense | diploid | 1 | U | Neutral | NA | 9874 |
| Missense | diploid | | U | Neutral | 0.43 | 33 |
| Missense | diploid | 1 | U | Low | 0.42 | 8210 |
| Missense | diploid | 1 | U | Low | 0.42 | 7951 |
| Missense | hetloss | | U | Low | 0.17 | 3213 |
| Missense | NA | | U | Low | NA | 21 |
| Missense | diploid | 1 | U | High | NA | 463 |
| Missense | diploid | 1 | V | High | NA | 468 |
| Missense | diploid | | U | Low | 0.8 | 1308 |
| Missense | diploid | 1 | U | Low | 0.21 | 121 |
| Missense | diploid | 1 | U | Low | NA | 151 |
| Missense | diploid | | U | Medium | 0.25 | 63 |
| Missense | diploid | 1 | U | Low | 0.98 | 71 |
| Missense | gain | | U | Neutral | 0.42 | 114 |
| Missense | diploid | | U | Low | 0.49 | 379 |
| Missense | diploid | 1 | U | Medium | 0.37 | 8620 |
| Missense | diploid | 1 | U | Medium | 0.37 | 8890 |
| Missense | hetloss | | U | Medium | NA | 4185 |
| Missense | NA | 1 | U | Neutral | NA | 451 |
| Missense | NA | 1 | U | Low | NA | 8 |
| Missense | diploid | 1 | U | Medium | 0.32 | 7801 |

| | | | | | | | |
|---|---|---|---|---|---|---|---|
| Missense | diploid | 1 | U | Medium | | 0.32 | 7553 |
| Missense | NA | 1 | U | Low | NA | | 193 |
| Missense | hetloss | | U | Neutral | | 0.19 | 778 |
| Missense | hetloss | | U | Neutral | | 0.19 | 771 |
| FS del | diploid | 7 | U | | | 0.46 | 105 |
| Nonsense | hetloss | 7 | U | | NA | | 4185 |
| Nonsense | diploid | 7 | U | | NA | | 33 |
| Nonsense | diploid | 7 | U | | NA | | 27 |
| Missense | diploid | | V | Neutral | NA | | 49 |
| Missense | diploid | | V | Neutral | NA | | 50 |
| Missense | diploid | 1 | U | Medium | | 0.35 | 741 |
| Missense | AMP | 1 | U | Medium | NA | | 4766 |
| Missense | gain | 1 | U | Medium | | 0.33 | 599 |
| Missense | diploid | | U | High | | 0.33 | 5317 |
| Missense | diploid | | U | Medium | | 0.35 | 825 |
| Missense | hetloss | 1 | U | Neutral | NA | | 733 |
| Missense | hetloss | 1 | U | Neutral | | 0.2 | 698 |
| Missense | diploid | | U | Low | | 1 | 276 |
| Missense | gain | | U | Low | NA | | 25 |
| Missense | diploid | | U | Low | | 0.47 | 39 |
| Missense | gain | 1 | U | Low | | 0.45 | 91 |
| Missense | diploid | | U | Neutral | | 0.46 | 57 |
| Missense | NA | | U | Low | NA | | 107 |
| Missense | diploid | 1 | U | Neutral | | 0.02 | 30 |
| Missense | diploid | 1 | U | Medium | | 0.18 | 277 |
| Missense | diploid | 1 | U | Medium | NA | | 311 |
| Missense | gain | | U | Low | | 0.3 | 484 |
| Missense | NA | | U | Neutral | NA | | 453 |
| Missense | diploid | | U | Low | | 0.5 | 3370 |
| FS ins | diploid | 7 | U | | NA | | 51 |
| FS ins | diploid | 7 | U | | NA | | 40 |
| Missense | diploid | 1 | U | Medium | | 0.33 | 338 |
| Missense | diploid | 1 | U | Medium | | 0.33 | 352 |
| Missense | NA | | U | Medium | NA | | 52 |
| FS del | diploid | 7 | U | | NA | | 50 |
| FS del | diploid | 7 | U | | NA | | 52 |
| Missense | hetloss | | U | Neutral | | 0.4 | 37 |
| Missense | diploid | | U | High | | 0.48 | 578 |
| Missense | NA | 1 | U | Medium | | 0.3 | 293 |
| Missense | diploid | | U | Low | | 0.44 | 74 |
| Missense | diploid | | U | Neutral | | 0.71 | 55 |

| | | | | | | | |
|---|---|---|---|---|---|---|---|
| Missense | diploid | 1 | U | Low | | 0.11 | 7951 |
| Missense | diploid | 1 | U | Low | | 0.11 | 8210 |
| Missense | diploid | | U | Low | | 0.1 | 37 |
| FS del | diploid | 7 | U | | | 0.92 | 34 |
| Missense | diploid | 1 | U | Low | | 0.39 | 7951 |
| Missense | diploid | 1 | U | Low | | 0.39 | 8210 |
| Missense | diploid | 1 | U | Low | NA | | 731 |
| Missense | diploid | 1 | V | Low | NA | | 727 |
| FS del | diploid | 7 | U | | | 0.39 | 326 |
| Missense | diploid | 1 | U | Low | | 0.21 | 8938 |
| Missense | diploid | 1 | U | Low | | 0.21 | 9187 |
| Missense | diploid | | U | Neutral | | 0.06 | 2314 |
| Missense | diploid | | U | Neutral | | 0.32 | 15124 |
| Missense | diploid | 1 | U | Neutral | | 0.11 | 1445 |
| Missense | diploid | 1 | U | Neutral | | 0.11 | 1463 |
| Missense | NA | 1 | U | Low | NA | | 2234 |
| Missense | diploid | | U | Neutral | | 0.19 | 1028 |
| Missense | NA | | U | | NA | | 1991 |
| Missense | diploid | 1 | U | Low | | 0.36 | 269 |
| Missense | diploid | 1 | U | Low | | 0.36 | 2521 |
| Missense | diploid | 1 | U | Low | | 0.36 | 2468 |
| Missense | diploid | | U | Neutral | | 0.53 | 18 |
| Missense | hetloss | | U | Low | | 0.5 | 1569 |
| Missense | diploid | 1 | V | Medium | NA | | 35 |
| Missense | diploid | 1 | U | Medium | NA | | 421 |
| Missense | diploid | 1 | U | Medium | | 0.23 | 414 |
| Missense | diploid | 1 | U | Neutral | NA | | 1810 |
| Missense | diploid | | U | Low | | 0.19 | 30 |
| Nonsense | diploid | 7 | U | | | 0.19 | 3370 |
| Missense | hetloss | 1 | U | Low | NA | | 186 |
| Missense | hetloss | 1 | U | Low | | 0.21 | 196 |
| Missense | diploid | | U | Medium | | 0.51 | 39 |
| Nonsense | diploid | 7 | U | | | 0.14 | 13534 |
| Nonsense | diploid | 7 | U | | | 0.24 | 5141 |
| Nonsense | diploid | 7 | U | | | 0.24 | 4994 |
| Missense | diploid | | U | Low | | 0.53 | 34 |
| Nonsense | diploid | 7 | U | | | 0.54 | 600 |
| Missense | gain | | U | Low | | 0.63 | 36 |
| Missense | NA | 1 | U | Low | NA | | 3226 |
| Missense | NA | 1 | U | Neutral | NA | | 1569 |

| | | | | | | | |
|---|---|---|---|---|---|---|---|
| Missense | diploid | | U | Neutral | | 0.68 | 157 |
| Missense | diploid | | U | Neutral | | 0.5 | 600 |
| Missense | diploid | | U | Neutral | | 0.5 | 216 |
| Missense | gain | | U | Neutral | | 0.15 | 436 |
| Missense | gain | | U | Neutral | | 0.15 | 425 |
| Missense | diploid | 3 | U | Neutral | | 0.03 | 573 |
| Missense | diploid | 3 | U | Neutral | | 0.5 | 8210 |
| Missense | diploid | 1 | U | Low | | 0.39 | 279 |
| Missense | diploid | 1 | U | Low | | 0.39 | 289 |
| Missense | hetloss | | U | Low | | 0.15 | 37 |
| Missense | diploid | | U | | | 0.15 | 232 |
| Missense | diploid | | U | Low | NA | | 9220 |
| Missense | hetloss | | U | Medium | | 1 | 56 |
| Missense | AMP | 1 | U | Medium | NA | | 4766 |
| Missense | diploid | 1 | U | Medium | | 0.58 | 741 |
| Missense | gain | 1 | U | Medium | | 0.65 | 599 |
| Missense | hetloss | | U | High | NA | | 91 |
| Missense | diploid | | U | Low | | 0.15 | 371 |
| Missense | gain | | U | Low | | 0.26 | 58 |
| Missense | diploid | | U | Neutral | | 0.07 | 180 |
| Missense | diploid | | U | Medium | | 0.09 | 935 |
| Missense | diploid | | U | Medium | | 0.08 | 617 |
| Missense | hetloss | 1 | U | Low | NA | | 4185 |
| Missense | diploid | 1 | V | Low | NA | | 9874 |
| Missense | diploid | 1 | V | Low | NA | | 9896 |
| Missense | NA | | U | Neutral | NA | | 253 |
| Missense | hetloss | 1 | U | Low | | 0.39 | 752 |
| Missense | hetloss | 1 | U | Low | | 0.39 | 819 |
| Missense | diploid | 1 | U | Low | NA | | 63 |
| Missense | diploid | 1 | U | Low | NA | | 48 |
| Missense | diploid | | U | Medium | | 0.41 | 249 |
| Missense | diploid | 1 | U | Medium | | 0.26 | 7226 |
| Missense | diploid | 1 | U | Medium | | 0.26 | 7027 |
| Missense | diploid | 1 | U | Medium | | 0.53 | 741 |
| Missense | AMP | 1 | U | Medium | NA | | 4766 |
| Missense | diploid | 1 | U | Medium | NA | | 1036 |
| Missense | diploid | 1 | U | Neutral | | 0.13 | 10489 |
| Missense | diploid | 1 | U | Neutral | | 0.13 | 10148 |
| Missense | diploid | 1 | U | Neutral | | 0.33 | 998 |
| Missense | gain | 1 | U | Neutral | | 0.22 | 1351 |
| Missense | diploid | | U | Neutral | | 0.29 | 15124 |

| | | | | | | | |
|---|---|---|---|---|---|---|---|
| Missense | diploid | | U | Medium | | 0.36 | 912 |
| Missense | diploid | | U | Medium | | 0.53 | 578 |
| Missense | hetloss | 1 | U | High | | 0.49 | 950 |
| Missense | diploid | | U | High | | 0.07 | 22 |
| Missense | gain | | U | Neutral | | 0.15 | 54 |
| Missense | diploid | 1 | U | Low | NA | | 2732 |
| Missense | diploid | | U | | | 1 | 75 |
| Missense | diploid | | U | Neutral | | 0.5 | 226 |

| SIFT | PROVEAN | PolyPhen-2 | MutationAss | SNV in dbSN | MAF(Minor Allel Frequency |
|---|---|---|---|---|---|
| Damaging | Neutral | ly/possibly dar | neutral | | |
| Damaging | Neutral | ly/possibly dar | neutral | | |
| Damaging | Deleterious | obably damagi | neutral | | |
| Damaging | Neutral | benign | neutral | | |
| Tolerated | Neutral | benign | neutral | **A324V C>T** | 0.348 |
| Damaging | Neutral | benign | neutral | | |
| Damaging | Neutral | benign | neutral | | |
| Tolerated | Neutral | benign | neutral | | |
| Tolerated | Neutral | benign | neutral | | |
| Tolerated | Neutral | benign | neutral | | |
| Tolerated | Neutral | benign | neutral | | |
| Tolerated | Neutral | benign | neutral | | |
| Damaging | Deleterious | bably damagi | neutral | | |
| Damaging | Deleterious | bably damagi | low | | |
| Damaging | Deleterious | bably damagi | low | | |
| Damaging | Deleterious | ly/possibly dar | low | | |
| Damaging | Deleterious | ly/possibly dar | low | | |
| Damaging | Deleterious | obably damagi | high | | |
| Damaging | Deleterious | obably damagi | high | | |
| Damaging | Deleterious | ssibly damagi | low | | |
| Damaging | Deleterious | benign | low | | |
| Damaging | Deleterious | benign | low | | |
| Damaging | Deleterious | possibly dama | medium | | |
| Damaging | Neutral | ossibly damagir | low | | |
| Damaging | Deleterious | benign | neutral | | |
| Damaging | Deleterious | bably damagi | low | | |
| Damaging | Deleterious | bably damagi | low | | |
| Damaging | Deleterious | bably damagi | low | | |
| Damaging | Deleterious | probably dama | medium | | |
| Tolerated | Neutral | benign | neutral | | |
| Tolerated | Neutral | benign | low | **E1016K 1 G>A** | NA |
| Damaging | Deleterious | bably damagi | medium | | |

| | | | | |
|---|---|---|---|---|
| Damaging | Deleterious | obably damagi | medium | |
| Damaging | Neutral | ossibly damagir | low | |
| Damaging | Neutral | obably damagi | neutral | |
| Damaging | Neutral | obably damagi | neutral | |
| | | | | |
| Damaging | Deleterious | obably damagi | neutral | |
| Damaging | Deleterious | obably damagi | neutral | |
| Damaging | Deleterious | ly/possibly dar | medium | |
| Damaging | Deleterious | ly/possibly dar | medium | |
| Damaging | Deleterious | ly/possibly dar | medium | |
| Damaging | Deleterious | probably dama | high | |
| Damaging | Deleterious | obably damagi | medium | |
| Tolerated | Deleterious | benign | neutral | |
| Tolerated | Deleterious | benign | neutral | |
| Damaging | Neutral | benign | low | |
| Tolerated | Neutral | benign | low | G363S, C>A |
| Damaging | Neutral | obably damagi | low | |
| Damaging | Neutral | robably damagi | low | |
| Damaging | Neutral | benign | neutral | |
| Damaging | Neutral | possibly dama | low | |
| Tolerated | Neutral | benign | neutral | |
| Damaging | Deleterious | robably damagi | medium | |
| Damaging | Deleterious | robably damagi | medium | |
| Damaging | Deleterious | benign | low | |
| Tolerated | Neutral | possibly dama | neutral | |
| Damaging | Neutral | obably damagi | low | |
| | | | | |
| Damaging | Deleterious | robably damagi | medium | |
| Damaging | Deleterious | robably damagi | medium | |
| Damaging | Neutral | probably dama | medium | |
| | | | | |
| Damaging | Neutral | obably damagi | neutral | |
| Damaging | Deleterious | obably damagi | high | |
| Tolerated | Deleterious | bly damaging/b | medium | |
| Damaging | Deleterious | probably dama | low | |
| Tolerated | Neutral | benign | neutral | |

| | | | | | |
|---|---|---|---|---|---|
| Damaging | Neutral | obably damagi | low | | |
| Damaging | Neutral | obably damagi | low | | |
| Damaging | Deleterious | obably damagi | low | | |
| Tolerated | Neutral | benign | low | L841F, C>T | |
| Tolerated | Neutral | benign | low | L841F, C>T | |
| Damaging | Neutral | obably damagi | low | | |
| Damaging | Neutral | obably damagi | low | | |
| Damaging | Neutral | ly/possibly da | low | | |
| Damaging | Neutral | ly/possibly da | low | | |
| Tolerated | Deleterious | ssibly damagi | neutral | | |
| Tolerated | Neutral | benign | neutral | | |
| Tolerated | Neutral | benign | neutral | | |
| Tolerated | Neutral | benign | neutral | | |
| Damaging | Deleterious | benign | low | | |
| Tolerated | Neutral | benign | neutral | | |
| Damaging | Deleterious | benign | neutral | | |
| Damaging | Deleterious | obably damagi | low | | |
| Damaging | Deleterious | obably damagi | low | | |
| Damaging | Deleterious | obably damagi | low | | |
| Damaging | Neutral | benign | neutral | P551L, C>T,2 | NA |
| Damaging | Deleterious | obably damagi | low | | |
| Damaging | Deleterious | obably damagi | low | | |
| Damaging | Deleterious | ly/possibly dar | medium | | |
| Damaging | Deleterious | ly/possibly dar | medium | | |
| Damaging | Neutral | ly/possibly dar | neutral | | |
| Tolerated | Neutral | benign | low | | |
| Tolerated | Neutral | benign | low | | |
| Tolerated | Neutral | benign | low | | |
| Damaging | Neutral | obably damagi | medium | Q74R,A>G,2 | NA |
| Damaging | Neutral | benign | low | | |
| Damaging | Neutral | benign | low | | |
| Damaging | Neutral | ly/possibly dar | low | | |
| Damaging | Neutral | bly damaging/b | neutral | | |

| | | | | | |
|---|---|---|---|---|---|
| Damaging | Neutral | benign | neutral | | |
| Damaging | Neutral | benign | neutral | | |
| Damaging | Neutral | benign | neutral | | |
| Damaging | Neutral | benign | neutral | | |
| Damaging | Neutral | benign | neutral | | |
| Tolerated | Neutral | benign | neutral | | |
| Tolerated | Neutral | benign | neutral | | |
| Damaging | Deleterious | robably damagi | low | | |
| Damaging | Deleterious | robably damagi | low | | |
| Damaging | Deleterious | probably dama | low | | |
| Damaging | Deleterious | obably damagi | medium | | |
| Tolerated | Deleterious | possibly dama | low | | |
| Damaging | Deleterious | obably damagi | medium | | |
| Damaging | Deleterious | obably damagi | medium | | |
| Damaging | Deleterious | obably damagi | medium | | |
| Damaging | Deleterious | obably damagi | medium | | |
| Damaging | Deleterious | probably dama | high | | |
| Damaging | Deleterious | obably damagi | low | | |
| Damaging | Deleterious | probably dama | low | | |
| Damaging | Deleterious | possibly dama | neutral | | |
| Damaging | Deleterious | probably dama | medium | | |
| Damaging | Deleterious | obably damagi | medium | | |
| Damaging | Neutral | benign | low | | |
| Damaging | Neutral | benign | low | **S33L 2 C>T** | NA |
| Damaging | Neutral | benign | low | **S33L 2 C>T** | NA |
| Damaging | Neutral | benign | neutral | | |
| Damaging | Neutral | benign | low | | |
| Damaging | Neutral | benign | low | | |
| Damaging | Neutral | ossibly damagir | low | | |
| Damaging | Neutral | ossibly damagir | low | | |
| Damaging | Deleterious | obably damagi | medium | | |
| Damaging | Neutral | benign | medium | S91L, C>T,2 | NA |
| Damaging | Neutral | benign | medium | S91L, C>T,3 | NA |
| Damaging | Deleterious | obably damagi | medium | | |
| Damaging | Deleterious | obably damagi | medium | | |
| Damaging | Deleterious | probably dama | medium | | |
| Damaging | Neutral | benign | neutral | | |
| Damaging | Neutral | benign | neutral | | |
| Damaging | Neutral | benign | neutral | | |
| Damaging | Neutral | benign | neutral | | |
| Tolerated | Deleterious | benign | neutral | | |

| | | | | |
|---|---|---|---|---|
| Damaging | Deleterious | obably damagi | medium | |
| Damaging | Deleterious | benign | medium | |
| Damaging | Deleterious | robably damagi | high | |
| Damaging | Deleterious | obably damagi | high | |
| Damaging | Deleterious | benign | neutral | |
| Damaging | Deleterious | benign | low | |
| Tolerated | Neutral | benign | neutral | |
| Tolerated | Neutral | benign | neutral | Y531H, T>C |

y) in dbSNP
