# Supplementary material for: Mutation analysis of large tumor suppressor genes LATS1 and LATS2 supports a tumor suppressor role in human cancer
Source: Protein Cell. 2014 Dec 9;6(1):6–11. doi: 10.1007/s13238-014-0122-4 (PMC4286129; doi:10.1007/s13238-014-0122-4)
Supplement: Supplementary file 4 — Supplementary material 4 (PDF 326 kb) [file 13238_2014_122_MOESM4_ESM.pdf]

GeneName	Transcript	SampleName	SampleID
LATS2/ENST00000382593	NM_014572	TCGA-F4-6570-01	1651303
LATS2/ENST00000382594	NM_014572	TCGA-73-4666-01	1780215
LATS2	NM_014572	TCGA-AG-A002-01	1651648
LATS2	NM_014572	NCI-H2126	687814
LATS2/ENST00000382593	NM_014572	TCGA-B5-A0JY-01	1783388
LATS2	NM_014572	TCGA-36-1568-01	1474996
LATS2/ENST00000382594	NM_014572	TCGA-BG-A0MU-01	1783437
LATS2/ENST00000382593	NM_014572	TCGA-AP-A056-01	1783334
LATS2	NM_014572	LUAD-5V8LT	1765104
LATS2/ENST00000382597	NM_014572	TCGA-AX-A0J1-01	1783377
LATS2/ENST00000382596	NM_014572	HCC37T	1919205
LATS2/ENST00000382595	NM_014572	ESO-114	1890886
LATS2/ENST00000382593	NM_014572	TCGA-AP-A0LM-01	1783352
LATS2	NM_014572	107430	1520444
LATS2	NM_014572	LUAD-YINHD	1765263
LATS2/ENST00000382598	NM_014572	TCGA-EW-A1P5-01	1900248
LATS2/ENST00000382597	NM_014572	TCGA-AZ-6601-01	1651175
LATS2/ENST00000382596	NM_014572	587284	1766769
LATS2/ENST00000382594	NM_014572	TCGA-05-4424-01	1780070
LATS2/ENST00000382593	NM_014572	TCGA-A1-A0SJ-01	1779257
LATS2_ENST00000382592	ENST0000038	TCGA-AO-A12B-01	1899791
LATS2/ENST00000382599	NM_014572	587342	1766789
LATS2/ENST00000382598	NM_014572	TCGA-D1-A103-01	1783494
LATS2/ENST00000382597	NM_014572	TCGA-38-4630-01	1780094
LATS2/ENST00000382596	NM_014572	587278	1766767
LATS2/ENST00000382595	NM_014572	TCGA-D1-A103-01	1783494
LATS2/ENST00000382594	NM_014572	LC_S12	1863723
LATS2/ENST00000382593	NM_014572	1_RESISTANT	2062378
LATS2	NM_014572	10	1481427
LATS2/ENST00000382595	NM_014572	TCGA-AN-A0XN-01	1779437
LATS2/ENST00000382594	NM_014572	TCGA-B5-A11J-01	1783404
LATS2/ENST00000382593	NM_014572	TCGA-18-3409-01	1780290
LATS2_ENST00000382592	ENST0000038	TCGA-BT-A20J-01	1779231
LATS2/LATS2/ENST00000382594	NM_014572	TCGA-46-3769-01	1781825
LATS2/LATS2/ENST00000382593	NM_014572	TARGET-30-PATHVK	1898456
LATS2	NM_014572	TCGA-08-0375-01	1337910
LATS2/ENST00000382593	NM_014572	TCGA-DM-A0XD-01	1651274
LATS2/ENST00000382594	NM_014572	TCGA-B5-A11E-01	1783399
LATS2_ENST00000382592	ENST0000038	TCGA-BT-A0YX-01	1779230
LATS2	NM_014572	TCGA-AG-A002-01	1651648

LATS2	NM_014572	P05-3852	1691471
LATS2/ENST00000382598	NM_014572	TCGA-A5-A0VQ-01	1783328
LATS2/ENST00000382597	NM_014572	1	1819807
LATS2/ENST00000382596	NM_014572	TCGA-CW-5589-01	1780010
LATS2/ENST00000382595	NM_014572	TCGA-55-1594-01	1780178
LATS2/ENST00000382594	NM_014572	ESO-0013	1890835
LATS2/ENST00000382593	NM_014572	TCGA-DM-A1DA-01	1651282
LATS2/ENST00000382594	NM_014572	TCGA-44-7670-01	1913986
LATS2/ENST00000382593	NM_014572	TCGA-B5-A11E-01	1783399
LATS2/ENST00000382601	NM_014572	TCGA-B0-5711-01	1779867
LATS2/ENST00000382600	NM_014572	HCT-15	1998443
LATS2/ENST00000382599	NM_014572	HCT-15	1998443
LATS2/ENST00000382598	NM_014572	TCGA-F4-6703-01	1651304
LATS2/ENST00000382597	NM_014572	TCGA-B5-A11E-01	1783399
LATS2/ENST00000382596	NM_014572	TCGA-44-6774-01	1780128
LATS2/ENST00000382594	NM_014572	1_PRE-TREATMENT	2062377
LATS2/ENST00000382595	NM_014572	1_RESISTANT	2062378
LATS2/ENST00000382593	NM_014572	TCGA-B5-A11E-01	1783399
LATS2	NM_014572	PD4120a	1331067
LATS2/ENST00000382593	NM_014572	ESO-717	1890958
LATS2	NM_014572	TCGA-AA-3525-01	1650985
LATS2/ENST00000382592	NM_014572	TCGA-59-2372-01	1731328
LATS2	NM_014572	TCGA-AA-3555-01	1651002
LATS2	NM_014572	Br27P	1312984
LATS2/ENST00000382594	NM_014572	TCGA-AA-3950-01	1651073
LATS2/ENST00000382593	NM_014572	TCGA-CM-5860-01	1651217
LATS2	NM_014572	PD0880a	753344
LATS2/ENST00000382596	NM_014572	TCGA-AP-A059-01	1783335
LATS2/ENST00000382595	NM_014572	TCGA-B5-A0JZ-01	1783389
LATS2/ENST00000382593	NM_014572	TCGA-CA-6717-01	1651188
LATS2/ENST00000382595	NM_014572	TCGA-64-5775-01	1780193
LATS2/ENST00000382594	NM_014572	TCGA-CA-6718-01	1651189
LATS2	NM_014572	16929	1423762
LATS2/ENST00000382597	NM_014572	TCGA-BS-A0UV-01	1783478
LATS2/ENST00000382596	NM_014572	HCT-15	1998443
LATS2/ENST00000382595	NM_014572	ccRCC-47	1980783
LATS2/ENST00000382593	NM_014572	TCGA-D1-A17Q-01	1783533
LATS2	NM_014572	SA214	1659984
LATS2/ENST00000382594	NM_014572	H1703	1870265
LATS2/ENST00000382593	NM_014572	TCGA-50-5044-01	1780151

AAMutation	CDSMutation	PrimaryTissue	TissueSubtype	TissueSubtype
p.R16Q	c.47G>A	Large intestin	Colon	Transverse
p.R16L	c.47G>T	Lung	NS	NS
p.S33L	c.98C>T	Large intestin	Rectum	NS
p.G40E	c.119G>A	Lung	NS	NS
p.D56Y	c.166G>T	Endometrium	NS	NS
p.P72L	c.215C>T	Ovary	NS	NS
p.I80fs*20	c.238delA	Endometrium	NS	NS
p.S91L	c.272C>T	Endometrium	NS	NS
p.Q105P	c.314A>C	Lung	NS	NS
p.A110T	c.328G>A	Endometrium	NS	NS
p.Q114R	c.341A>G	Liver	NS	NS
p.A120G	c.359C>G	Oesophagus	NS	NS
p.T168M	c.503C>T	Endometrium	NS	NS
p.S179L	c.536C>T	Breast	NS	NS
p.Y183C	c.548A>G	Lung	NS	NS
p.P190R	c.569C>G	Breast	NS	NS
p.E192K	c.574G>A	Large intestin	Colon	Sigmoid
p.P208L	c.623C>T	Large intestin	Colon	NS
p.G218V	c.653G>T	Lung	NS	NS
p.A324V	c.971C>T	Breast	NS	NS
p.G363S	c.1087G>A	Breast	NS	NS
p.R391H	c.1172G>A	Large intestin	Colon	NS
p.H393fs*6	c.1176_1177d	Endometrium	NS	NS
p.P414L	c.1241C>T	Lung	NS	NS
p.R415W	c.1243C>T	Large intestin	Colon	NS
p.A428V	c.1283C>T	Endometrium	NS	NS
p.P454L	c.1361C>T	Lung	NS	NS
p.P475L	c.1424C>T	NS	NS	NS
p.P479_A480insGR	c.1436_1437ir	Pancreas	NS	NS
p.P473_A480delPAPAPAPA	c.1416_1439d	Breast	NS	NS
p.A497T	c.1489G>A	Endometrium	NS	NS
p.P516L	c.1547C>T	Lung	NS	NS
p.R525C	c.1573C>T	Urinary tract	Bladder	NS
p.S528L	c.1583C>T	Lung	NS	NS
p.E550D	c.1650G>T	Autonomic ga	NS	NS
p.R558H	c.1673G>A	Central nervo	Brain	NS
p.R558L	c.1673G>T	Large intestin	Colon	Ascending
p.R558H	c.1673G>A	Endometrium	NS	NS
p.R558H	c.1673G>A	Urinary tract	Bladder	NS
p.A561T	c.1681G>A	Large intestin	Rectum	NS

p.G566C	c.1696G>T	Prostate	NS	NS
p.R581C	c.1741C>T	Endometrium	NS	NS
p.E591*	c.1771G>T	Kidney	NS	NS
p.S596R	c.1788C>G	Kidney	NS	NS
p.Q643E	c.1927C>G	Lung	NS	NS
p.K665R	c.1994A>G	Oesophagus	NS	NS
p.G675W	c.2023G>T	Large intestin	Caecum	NS
p.V682L	c.2044G>T	Lung	Right upper lo	NS
p.L693M	c.2077C>A	Endometrium	NS	NS
p.E722*	c.2164G>T	Kidney	NS	NS
p.E765D	c.2295G>T	Large intestin	Colon	NS
p.R769W	c.2305C>T	Large intestin	Colon	NS
p.F770L	c.2308T>C	Large intestin	Colon	Ascending
p.A773T	c.2317G>A	Endometrium	NS	NS
p.G803C	c.2407G>T	Lung	NS	NS
p.P838S	c.2512C>T	NS	NS	NS
p.P838S	c.2512C>T	NS	NS	NS
p.L841F	c.2521C>T	Endometrium	NS	NS
p.D852N	c.2554G>A	Breast	NS	NS
p.K863E	c.2587A>G	Oesophagus	NS	NS
p.A881V	c.2642C>T	Large intestin	Colon	Ascending
p.W896*	c.2688G>A	Ovary	NS	NS
p.L903I	c.2707C>A	Large intestin	Colon	Ascending
p.G909R	c.2725G>A	Central nervo	Brain	NS
p.C953G	c.2857T>G	Large intestin	Colon	Ascending
p.C953F	c.2858G>T	Large intestin	Colon	Ascending
p.C953*	c.2859C>A	Ovary	NS	NS
p.L967M	c.2899C>A	Endometrium	NS	NS
p.H970N	c.2908C>A	Endometrium	NS	NS
p.F978L	c.2934C>A	Large intestin	Colon	Ascending
p.P996L	c.2987C>T	Lung	NS	NS
p.D998G	c.2993A>G	Large intestin	Colon	Ascending
p.E1016K	c.3046G>A	Lung	NS	NS
p.E1039K	c.3115G>A	Endometrium	NS	NS
p.T1041I	c.3122C>T	Large intestin	Colon	NS
p.R1043L	c.3128G>T	Kidney	NS	NS
p.R1054*	c.3160C>T	Endometrium	NS	NS
p.E1067A	c.3200A>C	Breast	NS	NS
p.D1078Y	c.3232G>T	Lung	NS	NS
p.C1083Y	c.3248G>A	Lung	NS	NS

Histology	HistologySubt	HistologySubt	PubmedId	CGPStudy	SomaticStatus
Carcinoma	Adenocarcino	NS	-	COSU376	Variant of unk
Carcinoma	Adenocarcino	NS	-	COSU417	Variant of unk
Carcinoma	Adenocarcino	NS	-	COSU375	Confirmed So
Carcinoma	Non small cell	NS	16140923	COSU22; COSU	Confirmed So
Carcinoma	Endometrioid	NS	-	COSU419	Variant of unk
Carcinoma	Serous carcin	NS	21720365	COSU331	Confirmed So
Carcinoma	Endometrioid	NS	-	COSU419	Variant of unk
Carcinoma	Endometrioid	NS	-	COSU419	Variant of unk
Carcinoma	Adenocarcino	NS	22980975	COSU431	Variant of unk
Carcinoma	Endometrioid	NS	-	COSU419	Variant of unk
Carcinoma	NS	NS	-	COSU323	Variant of unk
Carcinoma	Adenocarcino	NS	23525077	COSU464	Variant of unk
Carcinoma	Endometrioid	NS	-	COSU419	Variant of unk
Carcinoma	HER-positive (	NS	20668451	COSU338	Confirmed So
Carcinoma	Adenocarcino	NS	22980975	COSU431	Variant of unk
Carcinoma	NS	NS	-	COSU414	Variant of unk
Carcinoma	Adenocarcino	NS	-	COSU376	Variant of unk
Carcinoma	Adenocarcino	NS	22895193	COSU452	Variant of unk
Carcinoma	Adenocarcino	NS	-	COSU417	Variant of unk
Carcinoma	NS	NS	-	COSU414	Variant of unk
Carcinoma	NS	NS	-	COSU414	Variant of unk
Carcinoma	Adenocarcino	NS	22895193	COSU452	Variant of unk
Carcinoma	Endometrioid	NS	-	COSU419	Variant of unk
Carcinoma	Adenocarcino	NS	-	COSU417	Variant of unk
Carcinoma	Adenocarcino	NS	22895193	COSU452	Variant of unk
Carcinoma	Endometrioid	NS	-	COSU419	Variant of unk
Carcinoma	Adenocarcino	NS	22975805	COSU453	Confirmed So
Malignant melanoma	NS	NS	24265154	COSU526	Confirmed So
Carcinoid-endocrine tur	Islet cell	NS	21252315	COSU333	Confirmed So
Carcinoma	NS	NS	-	COSU414	Confirmed So
Carcinoma	Endometrioid	NS	-	COSU419	Variant of unk
Carcinoma	Squamous cel	NS	-	COSU418	Variant of unk
Carcinoma	NS	NS	-	COSU413	Confirmed So
Carcinoma	Squamous cel	NS	-	COSU418	Variant of unk
Neuroblastoma	NS	NS	23334666	COSU466	Variant of unk
Glioma	Astrocytoma (	Glioblastoma	18772890	COSU473	Confirmed So
Carcinoma	Adenocarcino	NS	-	COSU376	Variant of unk
Carcinoma	Endometrioid	NS	-	COSU419	Previously Rep
Carcinoma	NS	NS	-	COSU413	Confirmed So
Carcinoma	Adenocarcino	NS	22810696	COSU375	Confirmed So

Carcinoma	NS	NS		22610119	COSU392	Variant of unk
Carcinoma	Endometrioid	NS	-		COSU419	Confirmed So
Carcinoma	Clear cell rena	NS		22397650	COSU439	Confirmed So
Carcinoma	Clear cell rena	NS	-		COSU416	Confirmed So
Carcinoma	Adenocarcino	NS	-		COSU417	Variant of unk
Carcinoma	Adenocarcino	NS		23525077	COSU464	Variant of unk
Carcinoma	Adenocarcino	NS	-		COSU376	Variant of unk
Carcinoma	Adenocarcino	NS	-		COSU417	Variant of unk
Carcinoma	Endometrioid	NS	-		COSU419	Variant of unk
Carcinoma	Clear cell rena	NS	-		COSU416	Confirmed So
Carcinoma	NS	NS		23856246	COSU504	Confirmed So
Carcinoma	NS	NS		23856246	COSU504	Confirmed So
Carcinoma	Adenocarcino	NS	-		COSU376	Variant of unk
Carcinoma	Endometrioid	NS	-		COSU419	Variant of unk
Carcinoma	Adenocarcino	NS	-		COSU417	Variant of unk
Malignant melanoma	NS	NS		24265154	COSU526	Confirmed So
Malignant melanoma	NS	NS		24265154	COSU526	Confirmed So
Carcinoma	Endometrioid	NS	-		COSU419	Variant of unk
Carcinoma	NS	NS		22722201	COSU385	Confirmed So
Carcinoma	Adenocarcino	NS		23525077	COSU464	Variant of unk
Carcinoma	Adenocarcino	NS	-		COSU376	Confirmed So
Carcinoma	Serous carcin	NS	-		COSU331	Variant of unk
Carcinoma	Adenocarcino	Mucinous	-		COSU376	Confirmed So
Glioma	Astrocytoma (	Glioblastoma		18772396	-	Confirmed So
Carcinoma	Adenocarcino	Mucinous	-		COSU376	Variant of unk
Carcinoma	Adenocarcino	NS	-		COSU376	Variant of unk
Carcinoma	Mucinous car	NS	-		COSU32; COS	Confirmed So
Carcinoma	Endometrioid	NS	-		COSU419	Variant of unk
Carcinoma	Endometrioid	NS	-		COSU419	Confirmed So
Carcinoma	Adenocarcino	Mucinous	-		COSU376	Variant of unk
Carcinoma	Adenocarcino	NS	-		COSU417	Variant of unk
Carcinoma	Adenocarcino	NS	-		COSU376	Variant of unk
Carcinoma	Adenocarcino	NS		18948947	COSU341	Confirmed So
Carcinoma	Endometrioid	NS	-		COSU419	Variant of unk
Carcinoma	NS	NS		23856246	COSU504	Confirmed So
Carcinoma	Clear cell rena	NS		23797736	COSU494	Confirmed So
Carcinoma	Endometrioid	NS	-		COSU419	Variant of unk
Carcinoma	Basal (triple-n	NS		22495314	COSU384	Confirmed So
Carcinoma	Squamous cel	NS		23033341	COSU456	Variant of unk
Carcinoma	Adenocarcino	NS	-		COSU417	Variant of unk

SampleSource	Zygosity	GenomicCo-ordinatesGRCh3	SIFT	PROVEAN	PolyPhen-2
Unknown	Heterozygous	13:21620119..21620119	Tolerated	Neutral	robably damagi
Tumour Samp	Unknown	13:21620119..21620119	Damaging	Deleterious	robably damagi
Tumour Samp	Unknown	13:21620068..21620068	Damaging	Neutral	benign
Cultured	Unknown	13:21620047..21620047	Damaging	Neutral	robably damagi
Tumour Samp	Heterozygous	13:21620000..21620000	Damaging	Deleterious	obably damagi
Tumour Samp	Heterozygous	13:21619951..21619951	Damaging	Deleterious	robably damagi
Tumour Samp	Heterozygous	13:21619928..21619928			
Tumour Samp	Heterozygous	13:21619894..21619894	Damaging	Neutral	benign
Tumour Samp	Unknown	13:21619852..21619852	Damaging	Neutral	bly/possibly dar
Tumour Samp	Heterozygous	13:21619838..21619838	Damaging	Neutral	ly/possibly dar
Tumour Samp	Heterozygous	13:21619825..21619825	Damaging	Neutral	possibly dama
Tumour Samp	Unknown	13:21565527..21565527	Damaging	Deleterious	robably damagi
Tumour Samp	Heterozygous	13:21563416..21563416	Damaging	Neutral	benign
Tumour Samp	Unknown	13:21563383..21563383	Damaging	Neutral	benign
Tumour Samp	Unknown	13:21563371..21563371	Damaging	Deleterious	benign
Tumour Samp	Heterozygous	13:21563350..21563350	Tolerated	Neutral	benign
Unknown	Heterozygous	13:21563345..21563345	Damaging	Neutral	benign
Tumour Samp	Unknown	13:21563296..21563296	Damaging	Deleterious	benign
Tumour Samp	Unknown	13:21563266..21563266	Tolerated	Deleterious	benign
Tumour Samp	Heterozygous	13:21562948..21562948	Tolerated	Neutral	benign
Tumour Samp	Heterozygous	13:21562832..21562832	Tolerated	Neutral	benign
Tumour Samp	Unknown	13:21562747..21562747	Damaging	Neutral	bly/possibly dar
Tumour Samp	Heterozygous	13:21562742..21562743			
Tumour Samp	Unknown	13:21562678..21562678	Damaging	Neutral	benign
Tumour Samp	Unknown	13:21562676..21562676	Damaging	Neutral	bly damaging/b
Tumour Samp	Heterozygous	13:21562636..21562636	Damaging	Neutral	benign
Tumour Samp	Unknown	13:21562558..21562558	Tolerated	Neutral	bly damaging/b
Unknown	Homozygous	13:21562495..21562495	Damaging	Neutral	possibly dama
Tumour Samp	Heterozygous	13:21562482..21562483			
Tumour Samp	Heterozygous	13:21562480..21562503			
Tumour Samp	Heterozygous	13:21562430..21562430	Tolerated	Neutral	benign
Tumour Samp	Unknown	13:21562372..21562372	Damaging	Deleterious	robably damagi
Tumour Samp	Unknown	13:21562346..21562346	Damaging	Neutral	bly damaging/b
Tumour Samp	Unknown	13:21562336..21562336	Damaging	Neutral	benign
Unknown	Unknown	13:21562269..21562269	Tolerated	Neutral	benign
Tumour Samp	Heterozygous	13:21562246..21562246	Tolerated	Neutral	benign
Unknown	Heterozygous	13:21562246..21562246	Tolerated	Neutral	benign
Tumour Samp	Heterozygous	13:21562246..21562246	Tolerated	Neutral	benign
Tumour Samp	Unknown	13:21562246..21562246	Tolerated	Neutral	benign
Tumour Samp	Unknown	13:21562238..21562238	Tolerated	Neutral	benign

Tumour Samp	Heterozygous	13:21562223..21562223	Tolerated	Neutral	benign
Tumour Samp	Unknown	13:21562178..21562178	Damaging	Deleterious	robably damagi
Tumour Samp	Unknown	13:21562148..21562148			
Tumour Samp	Unknown	13:21562131..21562131	Damaging	Neutral	ossibly damagir
Tumour Samp	Unknown	13:21557918..21557918	Tolerated	Neutral	benign
Tumour Samp	Unknown	13:21557851..21557851	Tolerated	Deleterious	bly damaging/b
Unknown	Heterozygous	13:21557822..21557822	Damaging	Deleterious	robably damagi
Tumour Samp	Unknown	13:21557801..21557801	Damaging	Deleterious	robably damagi
Tumour Samp	Heterozygous	13:21557768..21557768	Damaging	Neutral	obably damagi
Tumour Samp	Unknown	13:21557681..21557681			
Cultured	Heterozygous	13:21557550..21557550	Damaging	Deleterious	ly/possibly dar
Cultured	Heterozygous	13:21557540..21557540	Damaging	Deleterious	obably damagi
Unknown	Heterozygous	13:21557537..21557537	Damaging	Deleterious	robably damagi
Tumour Samp	Heterozygous	13:21557528..21557528	Damaging	Deleterious	obably damagi
Tumour Samp	Unknown	13:21557438..21557438	Damaging	Deleterious	robably damagi
Unknown	Homozygous	13:21555758..21555758	Tolerated	Deleterious	benign
Unknown	Homozygous	13:21555758..21555758	Tolerated	Deleterious	benign
Tumour Samp	Heterozygous	13:21555749..21555749	Tolerated	Neutral	benign
Tumour Samp	Unknown	13:21555716..21555716	Tolerated	Neutral	benign
Tumour Samp	Unknown	13:21555683..21555683	Damaging	Neutral	benign
Unknown	Unknown	13:21555628..21555628	Damaging	Deleterious	robably damagi
Tumour Samp	Heterozygous	13:21553914..21553914			
Unknown	Unknown	13:21553895..21553895	Damaging	Neutral	robably damagi
Tumour Samp	Heterozygous	13:21553877..21553877	Damaging	Deleterious	robably damagi
Unknown	Heterozygous	13:21549419..21549419	Damaging	Deleterious	oly/possibly dar
Unknown	Heterozygous	13:21549418..21549418	Damaging	Deleterious	robably damagi
Tumour Samp	Heterozygous	13:21549417..21549417			
Tumour Samp	Heterozygous	13:21549377..21549377	Damaging	Neutral	ly/possibly dar
Tumour Samp	Unknown	13:21549368..21549368	Damaging	Deleterious	robably damagi
Unknown	Heterozygous	13:21549342..21549342	Tolerated	Deleterious	benign
Tumour Samp	Unknown	13:21549289..21549289	Damaging	Deleterious	oly/possibly dar
Unknown	Heterozygous	13:21549283..21549283	Damaging	Deleterious	robably damagi
Unknown	Heterozygous	13:21549230..21549230	Tolerated	Neutral	benign
Tumour Samp	Heterozygous	13:21549161..21549161	Damaging	Deleterious	obably damagi
Cultured	Heterozygous	13:21549154..21549154	Damaging	Deleterious	obably damagi
Unknown	Unknown	13:21549148..21549148	Damaging	Deleterious	probably dama
Tumour Samp	Heterozygous	13:21549116..21549116			
Tumour Samp	Unknown	13:21549076..21549076	Damaging	Neutral	ossibly damagir
Cultured	Unknown	13:21549044..21549044	Damaging	Neutral	ossibly damagir
Tumour Samp	Unknown	13:21549028..21549028	Damaging	Deleterious	benign

MutationAss	SNV in dbSN	MAF(Minor Allel Frequency) in dbSNP
low		
medium		
low	**S33L 2 C>T**	NA
low		
low		
low		
medium	S91L, C>T,2	NA
neutral		
neutral		
neutral		
neutral		
neutral		
low		
low		
neutral	**P190R C>G**	0.0009
medium		
low		
neutral		
neutral	**A324V C>T**	0.348
low	G363S, C>A	
low		
neutral		
neutral		
neutral		
neutral		
low		
neutral		
low		
neutral		
low		
neutral		
neutral		
neutral		
neutral		
neutral		
neutral		

neutral		
low		
low		
low		
medium		
high		
high		
low		
medium		
medium		
low		
low		
medium		
neutral		
neutral		
low	L841F,C>T	
neutral		
neutral		
high		
low		
medium		
medium		
low		
low		
medium		
medium		
medium		
medium		
low	**E1016K 1 G>A**	NA
medium		
medium		
medium		
low		
low		
low		
