# Supplementary material for: Mutation analysis of large tumor suppressor genes LATS1 and LATS2 supports a tumor suppressor role in human cancer
Source: Protein Cell. 2014 Dec 9;6(1):6–11. doi: 10.1007/s13238-014-0122-4 (PMC4286129; doi:10.1007/s13238-014-0122-4)
Supplement: Supplementary file 5 — Supplementary material 5 (PDF 34 kb) [file 13238_2014_122_MOESM5_ESM.pdf]

Supplementary Table 1 Legend:

Detail information on non-synonymous mutations for human *LATS1* and *LATS2* genes collected in the COSMIC (http://cancer.sanger.ac.uk/cancergenome/projects/cosmic/) [1] and cBioPortal (http://http://www.cbioportal.org/public-portal/) [2] databases are presented.

LATS1 and **LATS2** data from the COSMIC database:

Column A: Gene name

Column B: Transcript

Column C: Sample name.

Column D: Sample ID/name

Column E: Amino acid mutation

Column F: Nucleotide change and position

Column G: Primary tissue

Column H: Tissue subtype

Column I: Tissue subtype

Column J: Histology

Column K: Histology subtype

Column L: Histology subtype

Column M: PubMed ID

Column N: CGP study

Column O: Somatic state

Column P: Sample source

Column Q: Zygosity

Column R-S: Genomic co-ordinates

Column T: The SIFT program (http://sift.jcvi.org/www/SIFT_enst_submit.html) [3]

Column U: PROVEAN (http://provean.jcvi.org/index.php) [4]

Column V: The PolyPhen-2 program (http://genetics.bwh.harvard.edu/pph2/) [5]

Column W: The Mutation Assessor program (http://mutationassessor.org/?set=tcga-gbm-nov.2009) [6]

Column X: Information in database on SNP's found in the same amino acid position

Column Y: Minor allele frequency in dbSNP

LATS1 and **LATS2** data from the cBioPortal database:

Column A: Cancer study

Column B: Type

Column C: Amino acid change

Column D: Amino acid position

Column E: Type of mutation

Column F: Copy number status of the mutated gene

Column G: 3D

Column H: Validation status

Column I: Allele frequency

Column J: Number of mutations in sample

Column K: The SIFT program (http://sift.jcvi.org/www/SIFT_enst_submit.html) [3]

Column L: PROVEAN (http://provean.jcvi.org/index.php) [4]

Column M: The PolyPhen-2 program (http://genetics.bwh.harvard.edu/pph2/) [5]

Column N: The Mutation Assessor program (http://mutationassessor.org/?set=tcga-gbm-nov.2009) [6]

Column O: Information in database on SNP's found in the same amino acid position

Column P: Minor allele frequency in dbSNP

References:

1. Forbes, S.A., Bindal, N., Bamford, S., Cole, C., Kok, C.Y., Beare, D., et al. 2010. COSMIC: mining complete cancer genomes in the Catalogue of Somatic Mutations in Cancer. Nucleic Acids Res. gkq929.
2. Gao, J., Aksoy, B.A., Dogrusoz, U., Dresdner, G., Gross, B., Sumer, S. O., et al., 2013. Integrative analysis of complex cancer genomics and clinical profiles using the cBioPortal. Science Signaling 6, pl1.
3. Ng, P.C., Henikoff, S. 2003. SIFT: Predicting amino acid changes that affect protein function. Nucleic Acids Res. 31**,** 3812-3814.
4. Choi, Y., Sims, G.E., Murphy, S., Miller, J.R., Chan, A.P. 2012. Predicting the functional effect of amino acid substitutions and indels. PLoS ONE 7, e46688.
5. Adzhubei, I.A., Schmidt, S., Peshkin, L., Ramensky, V.E., Gerasimova, A., Bork, P., Kondrashov, A.S., Sunyaev, S.R. 2010. A method and server for predicting damaging missense mutations. Nature Methods **7,** 248-249.
6. Reva, B., Antipin, Y., Sander, C. 2011. Predicting the functional impact of protein mutations: application to cancer genomics. Nucleic Acids Res. 39**,** e118.
